# *BMP2* rs1005464 is associated with mandibular condyle size variation

**DOI:** 10.1038/s41598-024-56530-3

**Published:** 2024-03-12

**Authors:** Guido Artemio Marañón-Vásquez, Mônica Tirre de Souza Araújo, Antônio Carlos de Oliveira Ruellas, Mírian Aiko Nakane Matsumoto, Marcio Figueiredo, Sandra Regina Santos Meyfarth, Lívia Azeredo Alves Antunes, Flares Baratto-Filho, Rafaela Scariot, Carlos Flores-Mir, Christian Kirschneck, Leonardo Santos Antunes, Erika Calvano Küchler

**Affiliations:** 1 Department of Pediatric Dentistry and Orthodontics, School of Dentistry, Federal University of Rio de Janeiro, Rua. Prof. Rodolpho Paulo Rocco, 325 – Cidade Universitária da Universidade Federal do Rio de Janeiro, Rio de Janeiro, RJ 21941-617 Brazil; 2 Department of Pediatric Dentistry, School of Dentistry of Ribeirão Preto, University of São Paulo, Avenida do Café, s/n., Ribeirão Preto, São Paulo 14040-904 Brazil; 3 Department of Specific Formation, School of Dentistry, Fluminense Federal University, Rua. Dr. Silvio Henrique Braune, 22 - Centro, Nova Friburgo, Rio de Janeiro 28625-650 Brazil; 4 Post-Graduation Program, Tuiuti University of Paraná, R. Padre Ladislau Kula, 395 - Santo Inácio, Curitiba, Brazil; 5 School of Dentistry, Univille – Univille – University of the Joinville Region, Rua Paulo Malschitzki, 10 - Zona Industrial Norte, Joinville, Santa Catarina 89219-710 Brazil; 6 Department of Stomatology, School of Dentistry, Federal University of Paraná, Av. Prefeito Lothário Meissner, 632 - Jardim Botânico, Curitiba, PR 80210-170 Brazil; 7 Graduate Orthodontic Program, School of Dentistry, Faculty of Medicine and Dentistry, University of Alberta, 5-528 Edmonton Clinic Health Academy, 11405 87 Ave NW, Edmonton, AB T6G 1C9 Canada; 8 Department of Orthodontics, Medical Faculty, University Hospital Bonn, Welschnonnenstr. 17, 53111 Bonn, Germany

**Keywords:** Genetic association study, Predictive markers, Cartilage, Oral anatomy

## Abstract

This study aimed to evaluate the association between single nucleotide polymorphisms (SNPs) in endochondral development-related genes and mandibular condyle shape, size, volume, and symmetry traits. Cone-beam Computed Tomographies and genomic DNA from 118 individuals were evaluated (age range: 15–66 years). Data from twelve 3D landmarks on mandibular condyles were submitted to morphometric analyses including Procrustes fit, principal component analysis, and estimation of centroid sizes and fluctuating asymmetry scores. Condylar volumes were additionally measured. Seven SNPs across *BMP2*, *BMP4*, *RUNX2* and *SMAD6* were genotyped. Linear models were fit to evaluate the effect of the SNPs on the mandibular condyles’ quantitative traits. Only the association between *BMP2* rs1005464 and centroid size remained significant after adjusting to account for the false discovery rate due to multiple testing. Individuals carrying at least one A allele for this SNP showed larger condylar size than common homozygotes GG (β = 0.043; 95% CI: 0.014—0.071; *P* value = 0.028). The model including *BMP2* rs1005464, age and sex of the participants explained 17% of the variation in condylar size. Shape, volume, and symmetry were not associated with the evaluated SNPs. These results suggest that *BMP2* rs1005464 might be associated with variation in the mandibular condyles size.

## Introduction

The mandibular condyle is the upper end of the condylar process of the mandible that originates from the already-formed intramembranous bone periosteum and grows by endochondral ossification, constituting an important growth site^1–3^. Although environmental factors influence the development of the mandibular condyle^3,4^; its morphogenesis, growth and homeostasis are determined primarily by intrinsic genetic factors^5,6^.

Genetic approach research has identified several relevant molecules for the development of the mandibular condyle^5,6^. The Runt-related transcription factor 2 (RUNX2) is essential for forming this structure; *Runx2*-deficient animals lack condylar cartilage^7^. This molecule is expressed at different locations and moments during the natural growth of condylar cartilage, regulating chondrocyte hypertrophy, cartilage matrix calcification, osteoblasts differentiation and osteoclasts function in endochondral ossification^8^. RUNX2 plays an important role in postnatal temporomandibular joint homeostasis by regulating chondrocyte-derived subchondral bone remodeling^9^. Bone morphogenetic proteins (BMPs), including BMP2 and BMP4, are also crucial for the growth of mandibular osteochondral tissues^10,11^. Specifically, BMP2 is further required for the postnatal maintenance of the mandibular condyle. BMP2 deletion causes breakage of the integrity of the condylar cartilage, accompanied by a decrease in its thickness, matrix synthesis, mineralization, chondrocyte proliferation, and increased expression of degenerative markers^10^. Mothers Against Decapentaplegic Homolog 6 (SMAD6) limits BMP signalling for proper bone development^12^. Although there is no specific evidence for mandibular condyle, this molecule would be relevant since it acts as a regulator of various stages of skeleton chondrogenesis, including anterior–posterior patterning, entry and exit of resting chondrocytes into the proliferative pool, and extracellular matrix synthesis^12^.

Evidence is limited regarding genes that contribute to normal variation in the configuration of the mandibular condyle in non-syndromic humans. A few genetic association studies have been performed indirectly evaluating the condyle as part of mandibular body and ramus measurements^13–16^, and only one evaluated condyle geometry through bidimensional imaging^17^. There are no studies evaluating the association between condylar phenotypes and single nucleotide polymorphisms (SNPs) in endochondral development-related genes. What do exist are studies showing that SNPs in genes involved in the aforesaid molecular processes are related to mandibular size and position phenotypes (*i.e.*, jaw sagittal and vertical relationships, mandibular retrognathism, mandibular length)^18–20^. Considering that condylar dimensions could influence mandibular traits^21,22^, it is reasonable to hypothesize that SNPs in endochondral development-related genes might primarily affect the configuration of the mandibular condyle.

Based on the above, this study aimed to explore the 3D phenotypic variation of the mandibular condyles and to evaluate the association of SNPs in *BMP2*, *BMP4*, *RUNX2* and *SMAD6* with shape, size, volume, and symmetry aspects of these structures.

## Results

### Method error

The intra-observer repeatability of the location of landmarks’ 3D coordinates and condylar volume measurements was high, with intraclass correlation coefficient ranging from 0.91–0.99. The measurement error for landmarks’ identification varied from 0.13–0.66 mm (X-axis: 0.20–0.66 mm; Y-axis: 0.17–0.46 mm; Z-axis: 0.13–0.44 mm), while for the right and left condylar volumes was 51.9 mm^3^ and 36.8 mm^3^, respectively. No proportion bias was identified by the Bland–Altman method for any of the measures assessed. Complete method error assessments are presented in Supplementary Tables S1 and S2.

### Genotyping quality control

The overall genotyping success rate was 100%. Allele frequencies for all the SNPs were in Hardy–Weinberg equilibrium, and the minor alleles showed a frequency above 15% (Table 1). There was 100% agreement between the duplicate genotyping and the original calls.

**Table 1:** Alleles and genotypes frequencies in the current sample.

Gene SNP (1/2)*	Genotyping rate	MAF	Genotypes n (%)			H-W *P* value
			Homozygous 1	Heterozygous	Homozygous 2	
*BMP2* rs1005464 (A/G)	100%	0.2288	7 (5.9)	40 (33.9)	71 (60.2)	0.912
*BMP2* rs235768 (A/T)	100%	0.3093	9 (7.6)	55 (46.6)	54 (45.8)	0.615
*BMP4* rs17563 (G/A)	100%	0.4153	16 (13.6)	66 (55.9)	36 (30.5)	0.257
*RUNX2* rs59983488 (T/G)	100%	0.1992	3 (2.5)	41 (34.7)	74 (62.7)	0.766
*RUNX2* rs1200425 (A/G)	100%	0.4576	25 (21.2)	58 (49.2)	35 (29.7)	0.994
*SMAD6* rs2119261 (T/C)	100%	0.3898	17 (14.4)	58 (49.2)	43 (36.4)	0.937
*SMAD6* rs3934908 (T/C)	100%	0.4407	21 (17.8)	62 (52.5)	35 (29.7)	0.774

SNP -single nucleotide polymorphism, MAF—minor allele frequency, H-W—Hardy Weinberg.

* (1 = minor allele / 2 = major allele).

### Main findings

The sample consisted of 118 participants (36 males and 82 females) with a mean age of 32.1 ± 14.4 years, presenting different skeletal malocclusions (Class I [0° < ANB < 4°]: n = 39, Class II [ANB ≥ 4°]: n = 45, Class III [ANB ≤ 0°]: n = 34).

**Figure 1:**
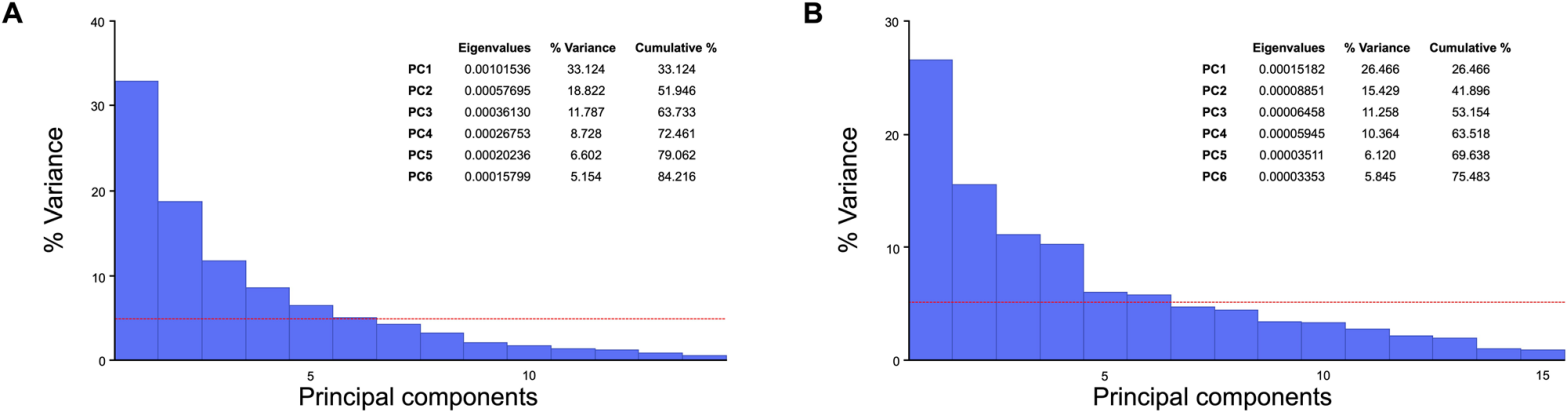
Scree plots show the variance explained by symmetric (**A**) and asymmetric (**B**) PCs.

**Figure 2:**
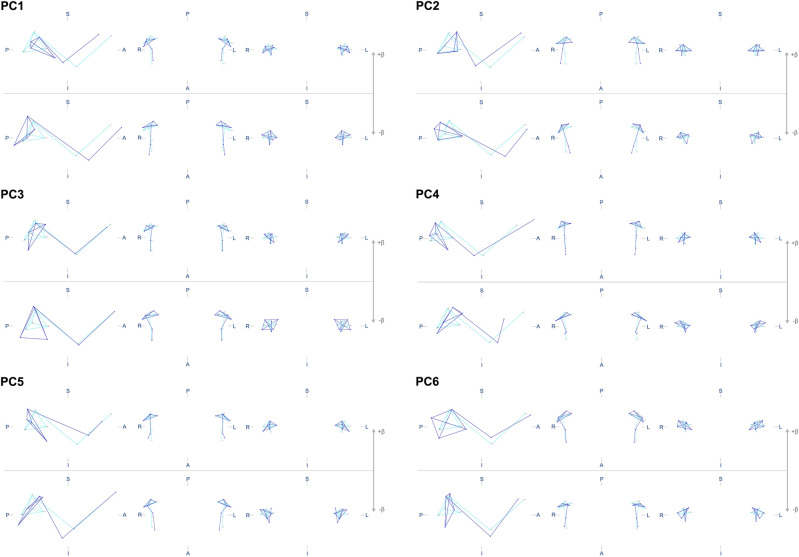
Mandibular condyles shape configurations of subjects with the most negative (− β) or positive (+ β) individual scores for symmetric PCs. S—superior, I—inferior, A—anterior, P—posterior, R—right, L—left. Light blue lines represent the average configuration, while dark blue lines represent the variation of interest.

**Figure 3:**
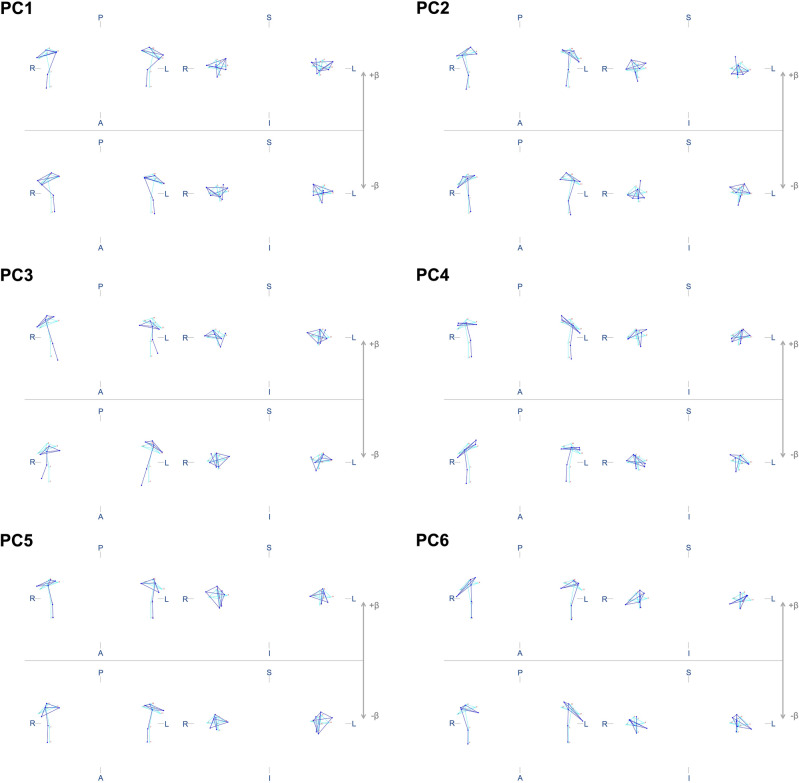
Mandibular condyles shape configurations of subjects with the most negative (− β) or positive (+ β) individual scores for asymmetric PCs. S—superior, I—inferior, A—anterior, P—posterior, R—right, L—left. Light blue lines represent the average configuration, while dark blue lines represent the variation of interest.

Six principal components (PC) were identified for each aspect of shape variation, symmetric and asymmetric, explaining a cumulative variance of 84.2% and 75.5%, respectively (Fig. 1). PC wireframes are shown in Figs. 2 and 3. Supplementary Table S3 describes the different shape configurations of the mandibular condyles for each PC, ranging from those that represented more negative and positive scores. Data on PC scores, centroid sizes and Mahalanobis shape fluctuating asymmetry scores showed an adequate distribution. The analyses showed an individual effect on the condylar size, shape, and volume (*P* < 0.001). Also, a side effect (right/left) on shape (*P* < 0.001) but not on condylar size (*P* = 0.230) and volume (*P* = 0.461) was observed.

The complete results of the implemented statistical models for genetic evaluations are presented in Supplementary Table S4. The following phenotype-genotype relationships were significant at the nominal level (*P* < 0.05; F test *P* value < 0.05; Table 2): *BMP2* rs1005464—symmetric PC2, symmetric centroid size, shape fluctuating asymmetry, and condylar volume; *BMP4* rs17563—shape fluctuating asymmetry; and *SMAD6* rs2119261—asymmetric PC4. The models for the effects of *BMP2* rs1005464 on symmetric centroid size and condylar volume showed the most significant explanatory power (adjusted R^2^: ~ 15–20%). The studied SNPs did not significantly influence the symmetric PC1, PC3–PC6; asymmetric PC1–PC3, PC5 and PC6; asymmetric centroid size; and right-left condylar volume difference.

**Table 2:** Single nucleotide polymorphisms with significant effects on mandibular condyles traits.

Mandibular condyles trait	Gene SNP (1/2)*	Genotypes	Model coefficients				B-H *P* value	Model fit measures		Power estimate
			β	95% CI		*P* value		F test *P* value	Adjusted R^2^	
				lower	upper					
Symmetric PC2	*BMP2* rs1005464 (A/G)	AG vs. GG	− 0.011	− 0.021	− 0.002	0.020	0.140	0.007	0.090	0.913
		AG + AA vs. GG	− 0.011	− 0.020	− 0.002	0.014	0.098	0.003	0.098	0.933
Asymmetric PC4	*SMAD6* rs2119261 (T/C)	CT vs. CC	− 0.003	− 0.006	0.000	0.030	0.210	0.009	0.084	0.525
Symmetric log centroid size	*BMP2* rs1005464 (A/G)	AG vs. GG	0.043	0.013	0.073	0.005	0.035	< 0.001	0.165	0.995
		AG + AA vs. GG	0.043	0.014	0.071	0.004	0.028	< 0.001	0.173	0.997
Mahalanobis shape FA scores	*BMP2* rs1005464 (A/G)	AA vs. GG	0.917	0.195	1.639	0.013	0.091	0.011	0.082	0.890
		AA vs. AG + GG	0.828	0.117	1.539	0.023	0.109	0.009	0.076	0.865
	*BMP4* rs17563 (G/A)	GG vs. AA	0.641	0.053	1.228	0.033	0.116	0.024	0.065	0.803
		GG vs. AG + AA	0.563	0.052	1.075	0.031	0.109	0.012	0.071	0.845
Condylar volume	*BMP2* rs1005464 (A/G)	AG vs. GG	235.6	43.0	428.2	0.018	0.126	< 0.001	0.191	0.996
		AG + AA vs. GG	232.9	50.8	415.0	0.014	0.098	< 0.001	0.192	0.997

SNP—single nucleotide polymorphism, CI—confidence interval, B-H—Benjamini–Hochberg procedure, PC—principal component, log—natural logarithm, FA—fluctuating asymmetry.

* (1 = minor allele / 2 = major allele).

Common homozygous genotypes were established as the reference category in the models.

The association between *BMP2* rs1005464 and symmetric centroid size was the only one that remained significant even after Benjamini–Hochberg adjustment (*P* ≤ 0.05; F test *P* value < 0.001; power = 0.99; Table 2). Individuals carrying at least one A allele for this variant showed significantly larger condylar size than GG common homozygotes (β = 0.043; 95% CI 0.014–0.071; adjusted *P* value = 0.028; F test *P* value < 0.001). Linear models, including *BMP2* rs1005464, age, and sex of participants as predictor variables, explained 17% of the variation in condylar size.

The age of the participants significantly influenced symmetric PC5 and PC6 (− β) and Mahalanobis shape fluctuating asymmetry scores (+ β), while sex had a significant effect on symmetric PC2 scores (+ β), asymmetric PC4 scores (− β), symmetric centroid sizes (+ β) and condylar volume (+ β) (Supplementary Table S5). Men showed condyles of greater size (β = 0.06; 95% CI 0.03–0.09; *P* value < 0.001) and volume (β = 435; 95% CI 235–635; *P* value < 0.001) than women. The variable skeletal malocclusion did not have a significant effect on any of the evaluated condylar traits (Supplementary Table S5).

## Discussion

Variations in the morphometric characteristics of the mandibular condyle might be involved in the development of skeletal malocclusions, occlusal disorders and joint dysfunction^23–27^. Little is known about the genetic basis of common craniofacial morphological variations. The complex background involved in the morphological determination of craniofacial phenotypes must be revealed. Understanding the role of genes in normal condyle development is the basis for exploring the molecular mechanisms involved in pathological conditions that affect the temporomandibular joint complex and craniofacial morphology. This is the first genetic association study that widely explored the phenotypic variability of this structure and evaluated its association with SNPs in endochondral development-related genes. Our findings suggest that *BMP2* rs1005464 would be associated with the size of the mandibular condyles.

Morphometric analyses revealed wide variation in the 3D configuration of the mandibular condyles. These structures were observed to present diverse shapes with variation in the width, length, and height of the mandibular condyle (neck and head, or only head) and different spatial orientations of the condylar head (*i.e.*, pitch, yaw, roll). Although complex, these data appear more informative than commonly used analyses on radiographs or bidimensional projections from cone beam computed tomographies (CBCT)^28^. No significant correlation was observed between the present study’s shape PC scores, size, and volume measures. These findings suggest that each condylar phenotype evaluated would provide relevant, different, and complementary information.

The mandibular condyle is subject to significant shape and size changes during active growth^29^, and even during adulthood^30^. This is due to its adaptive capacity that allows anatomical modification to ensure morphological, functional, and occlusal stability^31^. It has been reported that a rounded shape is more common in young adults, while the flat or angled shape is more observed in older people^32,33^. Although with several other complex changes added, our findings also showed that with the increase in age, the condylar head would adopt a more flattened configuration (*i.e.*, characteristics compatible with negative β for the PC5 symmetric component; Fig. 2).

In another aspect, the evidence is controversial regarding the influence of sex on condylar shape^30,32–35^; however, concerning to its size and volume, studies consistently show that men have larger condyles^21,36–39^. Our findings were in line with this previous evidence. Although the differences in shape between men and women were not evident, greater size and condylar volume were observed in males.

Regarding side effects, previous studies have shown that shape, size, and volume present differences between the right and left condyles^36,37,39,40^. In the present sample, only a side effect on the mandibular condyle shape was detected. We hypothesize that the right-left shape differences could be due to masticatory function occurring mainly on a preferred side^41^. The absence of right-left differences for volume and size could be because there were no individuals with evident facial skeletal asymmetries (chin deviation greater than 4 mm) in the present sample. Since some condylar traits could bring symmetry information not detected by other measures and considering that it has been reported that diverse condylar phenotypes are important determinants of mandibular configuration and facial morphology^22,42,43^, our data supports the need to carry out a 3D shape, size, and volume analysis for a better characterization of these structures.

Genetic analyses unveiled a significant association between *BMP2* rs1005464 and mandibular condyle size. BMPs are important growth factors that belong to the TGFβ superfamily with critical roles in skeletal development and chondrogenesis^44^. Deletion of BMP receptors in the embryonic or early postnatal periods results in non-development of secondary mandibular cartilages or attenuation of condylar cartilage extension, respectively^45–47^; demonstrating the importance that BMP signaling would have in the formation and growth of this structure. Specifically, BMP2 is known to participate in the formation of condensations, chondrocytes proliferation and differentiation, and extracellular matrix synthesis on articular tissues^44,48,49^. This molecule is highly expressed in the condylar cartilage anlagen^50,51^. It has been shown that adding exogenous BMP2 in condylar explants of *Runx2*-deficient animals induces chondrogenic differentiation, suggesting that this molecule would be an important factor for secondary cartilage development^50^. Moreover, BMP2 is required for postnatal maintenance of condylar cartilage integrity. Deletion of *Bmp2* in chondrocytes leads to early degeneration and decreased mineralization of the mandibular condyle, decreased cell proliferation, and increased expression of degenerative markers^10^. Based on this information, we assume that the association between rs1005464 and the mandibular condyle size is due to the influence of this variant on BMP2-mediated processes of formation, growth, and postnatal maintenance of condyle integrity.

rs1005464 has already shown a previous association with mandibular retrognathism and, in interaction with other SNPs in endochondral development genes, also contributed to presenting the dolichofacial pattern^19^; both features related to a small mandibular condyle. The study showed that GG homozygotes were more likely to have mandibular retrognathism^19^. Our findings aligned with this result, showing that GG individuals had smaller mandibular condyle centroid sizes. Considering that BMP2 participates not only in the endochondral ossification process but also in the intramembranous one^52^, it cannot be answered, based on our findings, if this variant influences only the size of the condyle or also contributes to the size of the mandibular body and rami. Unfortunately, several CBCTs analyzed did not fully include these structures (*i.e.*, mandibular body and rami), preventing this evaluation. It is important to mention that *BMP2* rs1005464 has additionally been associated with phenotypes like tooth size^53^ and dentoalveolar size discrepancies^54,55^, suggesting that it is likely that this variant has a pleiotropic effect influencing different size-related craniofacial features.

The present study has some limitations. Although the analyses were adjusted for the age and sex of the participants, and even though malocclusions did not influence the evaluated traits for the present study, the presence of selection bias due to other factors related to the study’s participants cannot be ruled out, since a convenience strategy for sampling and recruitment was implemented. In addition, since we worked with patients’ chart data, obtaining additional information to control for confounding factors was impossible. Considering that factors such as mandibular functionality, muscular activity, and static and dynamic occlusal contacts strongly influence the mandibular condyle^3,4,56^, these may have affected our results. Similarly, the participants’ body height could also have affected the results of condyle size and volume^13–16^.

Regarding another aspect, the number of SNPs evaluated was limited. SNPs in other genes crucially involved in mandibular condyle development (e.g., *SOX9*, *TGFβ*, *DLX5*, *SHOX2*, *FGFs*, *TWIST*1, *PTHrP* and *IHH*)^5,6^ need to be investigated in future studies. In addition, it should be emphasized that the absence of association of the other SNPs in *BMP4*, *RUNX2* and *SMAD6* does not mean that these genes are not involved in the configuration of the mandibular condyle; different variants in these genes could likely have a significant effect. On the other hand, some strengths should also be mentioned. Although the sample size is relatively small, the implemented models reached a power greater than 80% in most analyses. The reported *BMP2* rs1005464 association remains significant after applying the Benjamini–Hochberg adjustment, decreasing the probability that this result is a type 1 error. Another strength is the method implemented to assess the mandibular condyles, extracting the greatest possible information about its phenotypic variability.

## Methods

The present study followed an analytical observational cross-sectional design involving evaluating patient clinical records and subsequent analysis of phenotype-genotype relationships in eligible individuals. The Research Ethics Committee of the School of Dentistry of Ribeirao Preto, University of São Paulo, Brazil, approved and supervised the proper conduct of this study (n. 3.150.551). Research was conducted after approval of the Institutional Ethics Committee and all experiments were performed in accordance with regulations of the latest version of Declaration of Helsinki guidelines and its amendments. All participants and / or legal guardians gave written informed consent to participate in the study. Recommendations for Strengthening the Reporting of Genetic Association Studies were followed for the report^57^.

### Participants

Chart data of 403 patients from graduate and private dental clinics in two cities (Ribeirão Preto and Sorocaba), state of São Paulo, Brazil, with an indication for CBCT between 2008 and 2019, were screened to determine their eligibility. Ribeirão Preto and Sorocaba are in the southeastern region of Brazil. In this region, European ancestry predominates (60.7%), followed by African (32.0%) and Amerindian (7.3%)^58^.

CBCT from all individuals were taken for clinical purposes. Biologically unrelated individuals who had likely passed the peak of pubertal growth spurt of the jaws (≥ 15 years old)^59^, whose CBCT included both mandibular rami and condyles, were selected by a non-probabilistic convenience sampling. The exclusion criteria were diagnosed syndromes, dentofacial anomalies or metabolic diseases; history of facial trauma; degenerative temporomandibular joint disorders; parafunctional or chewing habits; previous orthopedic and / or ortho-surgical treatment; tooth loss affecting vertical dimension; and low-quality images.

After participant recruitment, 118 were considered eligible for the present study. Due to the relatively small number of participants, it was decided to analyze the total sample and retrospectively calculate the obtained power.

### Phenotyping

**Figure 4:**
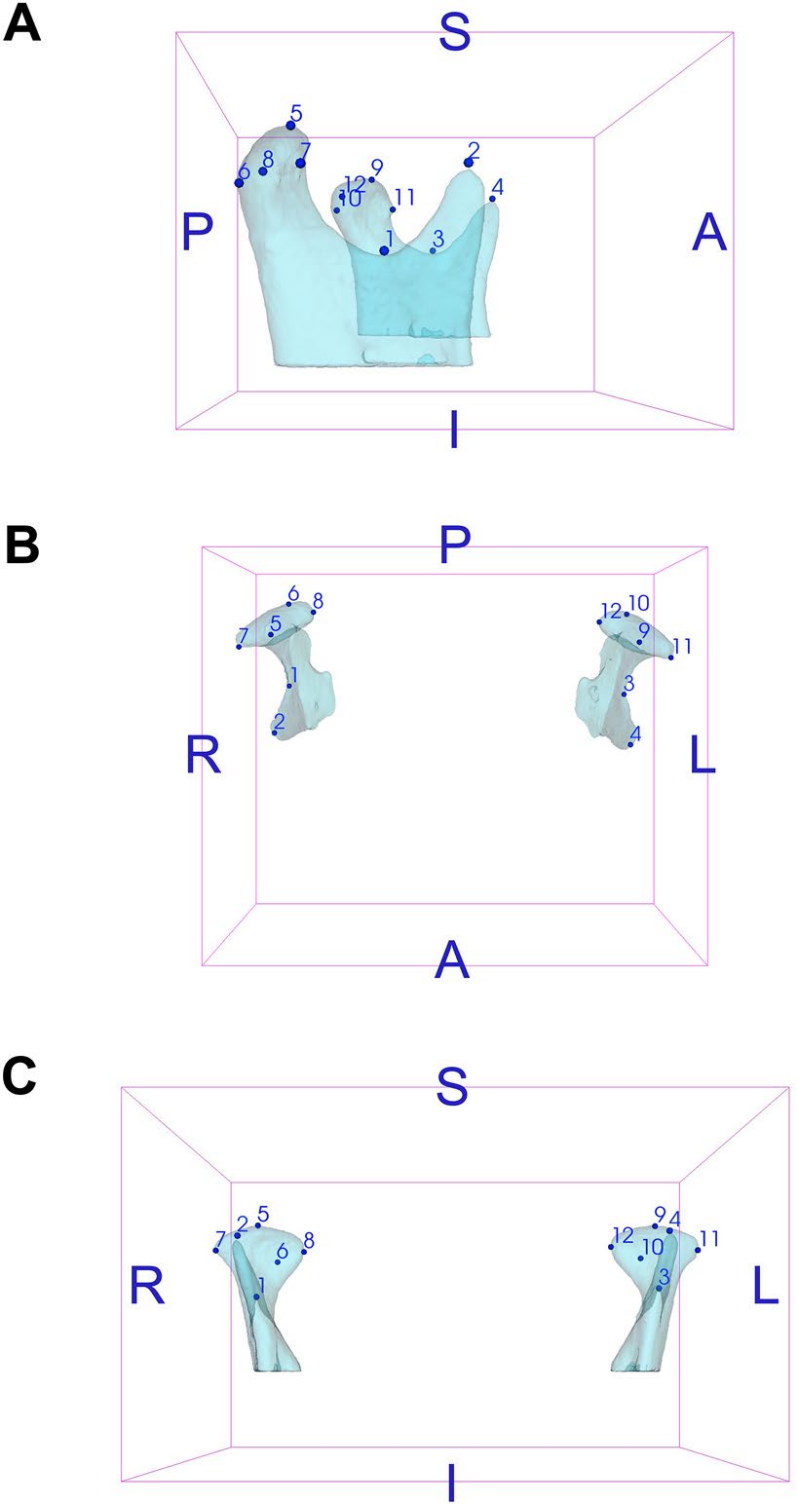
3D landmarks on the mandibular condyles. A (black letter)—right side view; B—top view; C—anterior view; S—superior; I—inferior; A (blue letter)—anterior; P—posterior; R—right; L—left; 1 and 3—right and left Sigmoid notch, respectively; 2 and 4—right and left Coronoid process, respectively; 5 and 9—right and left superior Condylion, respectively; 6 and 10—right and left posterior Condylion, respectively; 7 and 11—right and left lateral pole, respectively; 8 and 12—right and left medial pole, respectively.

**Figure 5:**
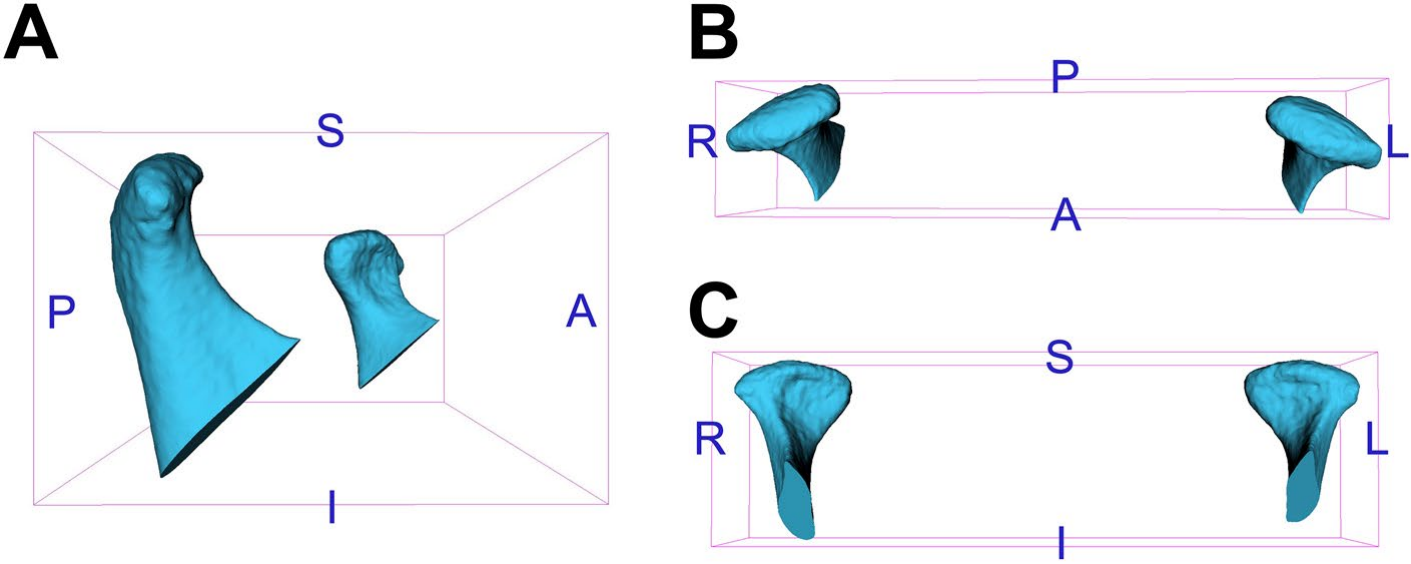
3D surface models of the mandibular condyles. A (black letter)—right side view; B—top view; C—anterior view; S—superior; I—inferior; A (blue letter)—anterior; P—posterior; R—right; L—left.

Participants’ CBCTs were evaluated for obtaining 3D landmark coordinates and condylar volume measurements. Preliminarily, CBCT files generated in Digital Imaging and Communications in Medicine format (.dcm files) were converted to Guys Imaging Processing Laboratory format (.gipl files) in ITK-SNAP 3.6.0 software (http://www.itksnap.org). Images were then resampled to 0.25 mm isotropic voxel size in 3D SLICER 5.0.3 software (http://www.slicer.org). Subsequently, the 3D analyses were performed following the step-by-step below:

1. Head orientation matching the midsagittal plane with Nasion, Crista Galli and Basion and the axial plane with the Frankfurt horizontal plane (Transforms module, 3D SLICER).
2. Construction of 3D volumetric label maps (segmentations) of approximately the upper two-thirds of the mandibular rami, including mandibular condyles and coronoid processes (Active Contour and Paintbrush modes, ITK-SNAP).
3. Identification and pre-labelling of 3D landmarks (Paintbrush mode, ITK-SNAP). Fourteen landmarks on the right and left condyles and surrounding structures were initially evaluated in the entire sample (Table 3). Anterior Condylion from both sides was excluded due to limitations during their identification. Hence, 12 landmarks were finally selected and included in further analyses (Fig. 4).
4. Generation of 3D surface models (.vtk files) of segmented structures and pre-labelled landmarks (Model Maker module, 3D SLICER).
5. Placement of definitive landmarks over pre-labelled landmarks on 3D surface models (Q3DC module, 3D SLICER).
6. Extraction of 3D coordinate data (X, Y and Z axes) corresponding to the landmarks (Markups module, 3D SLICER).
7. Cropping of the mandibular condyles for volume measurement. 3D surface models of the mandibular condyles were cropped at the level of an oriented plane, passing through the Sigmoid notch and the tip of the Coronoid process on the right and left sides (Easy Clip module, 3D SLICER) (Fig. 5).
8. Reconversion of cropped 3D surface ​​models to cropped volumetric label maps (Mesh to Label Map module, 3D SLICER).
9. Measurement of the volume (mm^3^) of the mandibular condyles using the cropped segmentations (ITK-SNAP).

**Table 3:** Definition of 3D landmarks.

Anatomical region	Landmark	Location
Mandibular condyle	Superior Condilion (SCo)*	The most superior point from the line connecting the lateral and medial poles of the mandibular condyle
	Posterior Condilion (PCo)*	The most posterior point from the line connecting the lateral and medial poles of the mandibular condyle
	Anterior Condilion (ACo)*	The most anterior point from the line connecting the lateral and medial poles of the mandibular condyle
	Lateral pole (LP)*	The most lateral point of the mandibular condyle
	Medial pole (MP)*	The most medial point of the mandibular condyle
Regions close to the mandibular condyle	Sigmoid notch (SN)*†	Deepest point of the mandibular Sigmoid notch
	Coronoid process (CP)*†	The most superior point of the Coronoid process of the mandible

*Paired landmarks located on the right and left sides.

^†^Landmarks used for delimitation of the mandibular condyles for volume measurement.

The aforementioned procedures were carried out by a single evaluator (GAMV) previously trained and calibrated by a specialized researcher (ACOR) with expertise in the described method^60^. Twenty percent of the sample was randomly selected to evaluate the method error. The same evaluator identified the 12 selected landmarks, extracted 3D coordinates, and measured the condylar volume again in this subsample after at least two weeks. Intra-observer repeatability was evaluated by the intraclass correlation coefficient. Random and systematic errors were assessed by Dahlberg’s Formula and by estimating the proportion bias according to the Bland–Altman method, respectively.

The 3D coordinates of the landmarks were imported into MORPHOJ 1.07a software (https://morphometrics.uk/MorphoJ_page.html) to conduct geometric morphometric analyses. These assessments were performed under two symmetry perspectives^61,62^: (a) object symmetry for shape evaluations (*i.e.*, considering the relative disposition of the condyles to each other since the position of each condyle is an integral aspect of mandibular symmetry), and (b) matching symmetry for condylar size evaluations (*i.e.*, disregarding the relative disposition of the condyles to each other, analyzing similarities and differences between both structures).

**Figure 6:**
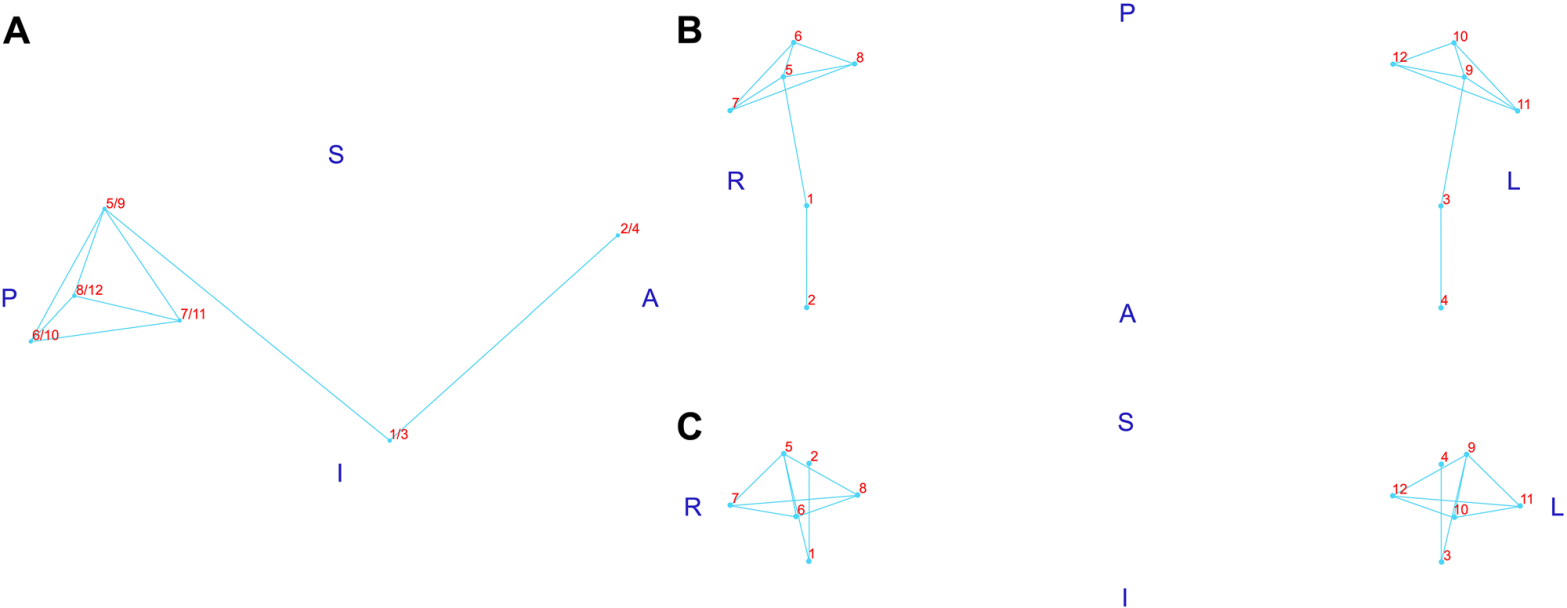
Wireframe of the mean configuration of 3D landmarks on the mandibular condyles. A (black letter)—X versus Y axis (side view); B—X versus Z axis (top view); C—Y versus Z axis (anterior view); S—superior; I—inferior; A (blue letter)—anterior; P—posterior; R—right; L—left; 1 and 3—right and left Sigmoid notch, respectively; 2 and 4—right and left Coronoid Process, respectively; 5 and 9—right and left superior Condylion, respectively; 6 and 10—right and left posterior Condylion, respectively; 7 and 11—right and left lateral pole, respectively; 8 and 12—right and left medial pole, respectively.

The following geometric morphometric analyses were conducted:

1. Full Procrustes fit to extract the shape variation of the data set. The analysis under object symmetry generated two data matrices for the symmetric and asymmetric components of variation. In contrast, the analysis under matching symmetry generated a data matrix containing the Procrustes coordinates.
2. Generation of covariance matrices and subsequent PC Analysis to identify the most critical aspects of variation in the data sets (*i.e.*, those explaining at least 5% of the variation). Individual scores were generated for the identified PCs. Wireframes were created to represent the mean configuration and deformation of the structures of interest (Fig. 6).
3. Condylar size estimation via centroid size. As mentioned above, the analysis was performed under the matching symmetry perspective, where a data set was generated based on the individual means of the right and left condyles.
4. Estimation of the condylar size asymmetry via asymmetric centroid size.
5. Estimating individual scores of shape fluctuating asymmetry (*i.e.*, deviations from the mean asymmetry) in units of Mahalanobis distances (dimensioned concerning the variation of the sample asymmetry, independently of the directional asymmetry).
6. Procrustes ANOVA to assess individual and side (right / left) effects on the condylar size and 3D shape variation.

### Genotyping

Saliva samples containing squamous epithelial cells from the buccal mucosa were collected to obtain genomic DNA. The participants were asked to rinse their mouth with 5 ml of 5% saline solution for 60 s and expectorate the volume into 15 ml propylene tubes (CORNING Inc., Corning, NY, USA). Next, sterile disposable cytobrushes (Plus GT, Medscand, COOPERSURGICAL Inc., Trumbull, CT, USA) were swiped and rolled twice to three times at the tongue’s base and inner surface of the cheek on the right and left sides to collect additional biological material. The tubes containing the samples were centrifuged at 550 rpm for 10 min to sediment the cell pellet. The supernatant was discarded, and the pellet was resuspended in 1 ml of extraction buffer (Tris–HCl 10 mM, pH 7.8; EDTA 5 mM; SDS 0.5%). The samples were stored at − 20 °C until processing.

Genomic DNA was extracted from buccal cells following a previously reported method^63^. The concentration and purity of the extracted DNA were evaluated using a spectrophotometer (NanoDrop 1000, THERMO SCIENTIFIC Inc., Waltham, MA, USA). Seven single nucleotide polymorphisms (SNPs) across four genes (*Bone Morphogenetic Protein 2* [*BMP2*], *Bone Morphogenetic Protein 4* [*BMP4*], *Runt-Related Transcription Factor 2* [*RUNX2*], and *SMAD Family Member 6* [*SMAD6*]) were chosen based on a minor allele frequency > 5% by searching public databases, previously reported association with craniofacial phenotypes, and their location on genes with biological implications in the growth, development and maintenance of the mandibular condyles (Table 4). Genotyping was blindly performed by polymerase chain reactions (PCR) using endpoint analysis^64^ and TaqMan technology on a real-time PCR system (Step One Plus Real-Time PCR System, APPLIED BIOSYSTEMS, Foster City, CA, USA). Primers, probes, and universal master mix were provided by APPLIED BIOSYSTEMS (Foster City, CA, USA). Ten percent of the sample was genotyped in duplicate to test the quality of the process.

**Table 4:** Single nucleotide polymorphisms studied.

Gene	Band	Position (GRCh37)	Reference sequence	Functional consequence	Base change (Context sequence)	MAF (ALFA)
*BMP2*	20p12.3	6756148	rs1005464	Intron variant	ATC[A/G]CCT	A = 0.2194
*BMP2*	20p12.3	6759115	rs235768	Missense variant*	CAG[A/T]CTT	A = 0.3678
*BMP4*	14q22.2	54417522	rs17563	Missense variant**	ATC[A/G]CCT	A = 0.4803
*RUNX2*	6p21.1	45295722	rs59983488	Intron variant	GGG[G/T]AGT	T = 0.0710
*RUNX2*	6p21.1	45527132	rs1200425	Intron variant	TTT[A/G]GAA	A = 0.3891
*SMAD6*	15q22.31	67011980	rs2119261	Intron variant	CTC[C/T]ATG	T = 0.3700
*SMAD6*	15q22.31	67061467	rs3934908	Intron variant	AAG[C/T]CCT	T = 0.4498

MAF—minor allele frequency, ALFA—Allele Frequency Aggregator (NCBI database of Genotypes and Phenotypes [dbGaP]).

* Arg → Ser; ** Val → Ala.

Sources of information: dbSNP from: http://www.ncbi.nlh.nih.gov/snp/; http://genome.uscs.edu/; e, https://www.thermofisher.com.

### Statistical analysis

Descriptive statistics were used to report participant characteristics, and alleles and genotypes distributions for each SNP. Hardy–Weinberg equilibrium was evaluated by applying the chi-square test (or chi-square with Yates’s correction, when necessary). The distribution and normality of the phenotype data were assessed using histograms, the Shapiro–Wilk test, and skewness values. ANOVA evaluated individual and side (right/left) effects on condylar volume measures. Univariate linear regressions were used to evaluate the single effect of the variables sex, age, and malocclusion on the mandibular condylar traits (Omnibus ANOVA test).

Linear models were fitted by the method of ordinary least squares to evaluate the effect of the SNPs studied on the following quantitative traits: individual scores for PCs of shape variation (symmetric and asymmetric components), symmetric and asymmetric centroid sizes, shape fluctuating asymmetry scores, the volume of the mandibular condyles, and right-left condylar volume difference. Since the univariate linear regressions detected an effect of the variables sex and age on some of the studied condylar traits, these were included as covariates in the models. Additional analyses were performed in dominant and recessive models for the lower-frequency alleles.

The assumptions of normality of residuals, homogeneity of residual variances and multicollinearity for the implemented models were verified by the Shapiro–Wilk test, Levene’s test and the Variance Inflation Factor, respectively. The F omnibus test was used to evaluate the fit of the models and the R^2^ value to measure how much the predictor variables (*i.e.*, SNPs, sex, and age) explained the variation of quantitative traits.

A *P* value less than or equal to 0.05 was established as significant at the nominal level. The Benjamini–Hochberg procedure adjusted the *P* values to account for the false discovery rate due to multiple testing. All the mentioned analyses were conducted in JAMOVI 2.3.18.0 software (https://www.jamovi.org/). Post hoc estimates of the power achieved by the analyses were performed in G*POWER 3.1.9.6 (https://www.psychologie.hhu.de/arbeitsgruppen/allgemeine-psychologie-und-arbeitspsychologie/gpower) based on the significance level, sample size, and effect size f^2^ estimated by the models. The effect size was determined by imputating ρ^2^ coefficients from predictor variables correlation.

### Supplementary Information

Supplementary Information 1.

Supplementary Tables.

## Data Availability

All data generated or analyzed during this study are included in this published article and its supplementary files. Supplementary files contain tables with method error evaluations, description of the principal components, and the complete statistical analyses performed. In addition, an .xlsx file with the raw datasets used in the analyses are provided.

## References

[1] Koski K (1968). Cranial growth centers: Facts or fallacies?. Am. J. Orthod..

[2] Moss ML, Rankow RM (1968). The role of the functional matrix in mandibular growth. Angle Orthod..

[3] Petrovic AG (1972). Mechanisms and regulation of mandibular condylar growth. Acta Morphol. Neerl. Scand..

[4] Duterloo, H. S. & Wolters, J. M. Experiments on the significance of articular function as a stimulating chondrogenic factor for the growth of secondary cartilages of the rat mandible. *Trans. Eur. Orthod. Soc*. 103–115 (1971). 5293075

[5] Hinton RJ (2014). Genes that regulate morphogenesis and growth of the temporomandibular joint: A review. Dev. Dyn..

[6] Hinton RJ, Jing J, Feng JQ (2015). Genetic influences on temporomandibular joint development and growth. Curr. Top. Dev. Biol..

[7] Shibata S (2004). Runx2-deficient mice lack mandibular condylar cartilage and have deformed Meckel’s cartilage. Anat. Embryol..

[8] Rabie ABM, Tang GH, Hägg U (2004). Cbfa1 couples chondrocytes maturation and endochondral ossification in rat mandibular condylar cartilage. Arch. Oral Biol..

[9] Liao L (2019). Deletion of Runx2 in condylar chondrocytes disrupts TMJ tissue homeostasis. J. Cell. Physiol..

[10] O’Brien MH, Dutra EH, Mehta S, Chen P, Yadav S (2021). BMP2 is required for postnatal maintenance of osteochondral tissues of the temporomandibular joint. Cartilage.

[11] Semba I (2000). Positionally-dependent chondrogenesis induced by BMP4 is co-regulated by Sox9 and Msx2. Dev. Dyn..

[12] Estrada KD, Retting KN, Chin AM, Lyons KM (2011). Smad6 is essential to limit BMP signaling during cartilage development. J. Bone Miner. Res..

[13] Tomoyasu Y (2009). Further evidence for an association between mandibular height and the growth hormone receptor gene in a Japanese population. Am. J. Orthod. Dentofacial Orthop..

[14] Yamaguchi T, Maki K, Shibasaki Y (2001). Growth hormone receptor gene variant and mandibular height in the normal Japanese population. Am. J. Orthod. Dentofacial Orthop..

[15] Zhou J (2005). The growth hormone receptor gene is associated with mandibular height in a Chinese population. J. Dent. Res..

[16] Kang EH (2009). Association of the growth hormone receptor gene polymorphisms with mandibular height in a Korean population. Arch. Oral Biol..

[17] Constant M (2017). Condylar geometry variation is associated with ENPP1 variant in a population of patients with dento-facial deformities. J. Craniomaxillofac. Surg..

[18] Olsson B (2021). Single nucleotide polymorphisms in Runt-related Transcription Factor 2 and Bone Morphogenetic Protein 2 impact on their maxillary and mandibular gene expression in different craniofacial patterns—A comparative study. Ann. Maxillofac. Surg..

[19] Küchler EC (2021). Potential interactions among single nucleotide polymorphisms in bone- and cartilage-related genes in skeletal malocclusions. Orthod. Craniofac. Res..

[20] Hussein AS, Porntaveetus T, Abid M (2022). The association of polymorphisms in BMP2/MYO1H and skeletal Class II div.1 maxillary and mandibular dimensions. A preliminary report. Saudi J. Biol. Sci..

[21] Saccucci M (2012). Condylar volume and condylar area in class I, class II and class III young adult subjects. Head Face Med..

[22] You K, Lee K, Lee S, Baik H (2010). Three-dimensional computed tomography analysis of mandibular morphology in patients with facial asymmetry and mandibular prognathism. Am. J. Orthod. Dentofacial Orthop..

[23] Burke G, Major P, Glover K, Prasad N (1998). Correlations between condylar characteristics and facial morphology in Class II preadolescent patients. Am. J. Orthod. Dentofacial Orthop..

[24] Katsavrias EG, Halazonetis DJ (2005). Condyle and fossa shape in Class II and Class III skeletal patterns: A morphometric tomographic study. Am. J. Orthop. Dentofacial Orthop..

[25] Chang MS (2018). Relationships between temporomandibular joint disk displacements and condylar volume. Oral Surg. Oral Med. Oral Pathol. Oral Radiol..

[26] Kurita H, Ohtsuka A, Kobayashi H, Kurashina K (2002). Alteration of the horizontal mandibular condyle size associated with temporomandibular joint internal derangement in adult females. Dentomaxillofac. Radiol..

[27] Seo BY, An JS, Chang MS, Huh KH, Ahn SJ (2020). Changes in condylar dimensions in temporomandibular joints with disk displacement. Oral Surg. Oral Med. Oral Pathol. Oral Radiol..

[28] Arayapisit T (2023). Understanding the mandibular condyle morphology on panoramic images: A conebeam computed tomography comparison study. Cranio.

[29] Karlo CA (2010). Size, shape and age-related changes of the mandibular condyle during childhood. Eur. Radiol..

[30] Rodrigues VP (2019). Tooth loss and craniofacial factors associated with changes in mandibular condylar morphology. Cranio.

[31] Alomar X (2007). Anatomy of the temporomandibular joint. Semin. Ultrasound CT. MR..

[32] Tassoker M, Kabakci AD, Akin D, Sener S (2017). Evaluation of mandibular notch, coronoid process, and mandibular condyle configurations with cone beam computed tomography. Biomed. Res..

[33] Yalcin ED, Ararat E (2019). Cone-beam computed tomography study of mandibular condylar morphology. J. Craniofac. Surg..

[34] Yale SH, Allison BD, Hauptfuehrer JD (1966). An epidemiological assessment of mandibular condyle morphology. Oral Surg. Oral Med. Oral Pathol..

[35] Coogan JS, Kim D, Bredbenner TL, Nicolella D (2018). Determination of sex differences of human cadaveric mandibular condyles using statistical shape and trait modelling. Bone.

[36] Tecco S (2010). Condylar volume and surface in Caucasian young adult subjects. BMC Med. Imaging.

[37] Safi A (2018). Volumetric analysis of 700 mandibular condyles based upon cone beam computed tomography. J. Craniofac. Surg..

[38] Safi A (2018). Age-related volumetric changes in mandibular condyles. J. Craniofac. Surg..

[39] Lentzen M (2022). A volumetric study of mandibular condyles in orthognathic patients by semiautomatic segmentation. Oral Maxillofac. Surg..

[40] Rodrigues AF, Fraga MR, Vitral RWF (2009). Computed tomography evaluation of the temporomandibular joint in Class I malocclusion patients: Condylar symmetry and condyle-fossa relationship. Am. J. Orthod. Dentofacial Orthop..

[41] Dias GJ, Cook RB, Mirhosseini M (2011). Influence of food consistency on growth and morphology of the mandibular condyle. Clin. Anat..

[42] Oh M, Cho J (2020). The three-dimensional morphology of mandible and glenoid fossa as contributing factors to menton deviation in facial asymmetry-retrospective study. Prog. Orthod..

[43] Alam MK (2021). A 3D cone beam computed tomography (CBCT) investigation of mandibular condyle morphometry: Gender determination, disparities, asymmetry assessment and relationship with mandibular size. Saudi Dent. J..

[44] Chen D, Zhao M, Mundy GR (2004). Bone morphogenetic proteins. Growth Factors.

[45] Gu S (2014). BMPR1A mediated signaling is essential for temporomandibular joint development in mice. PLoS One.

[46] Dudas M, Sridurongrit S, Nagy A, Okazaki K, Kaartinen V (2004). Craniofacial defects in mice lacking BMP type I receptor Alk2 in neural crest cells. Mech. Dev..

[47] Jing J (2014). Critical role of Bmpr1a in mandibular condyle growth. Connect. Tissue Res..

[48] Gamer LW (2018). The role of Bmp2 in the maturation and maintenance of the murine knee joint. J. Bone Miner. Res..

[49] Rountree RB (2004). BMP receptor signaling is required for postnatal maintenance of articular cartilage. PLoS Biol..

[50] Fukuoka H, Shibata S, Suda N, Yamashita Y, Komori T (2007). Bone morphogenetic protein rescues the lack of secondary cartilage in Runx2-deficient mice. J. Anat..

[51] Anderson HC, Hodges PT, Aguilera XM, Missana L, Moylan PE (2000). Bone morphogenetic protein (BMP) localization in developing human and rat growth plate, metaphysis, epiphysis, and articular cartilage. J. Histochem. Cytochem..

[52] Stoeger T (2002). In situ gene expression analysis during BMP2-induced ectopic bone formation in mice shows simultaneous endochondral and intramembranous ossification. Growth Factors.

[53] Gerber JT (2021). Odontogenesis-related candidate genes involved in variations of permanent teeth size. Clin. Oral Investig..

[54] Sharma K (2021). Single nucleotide polymorphisms of BMP2 gene association with skeletal Class I crowding: A PCR study. J. Contemp. Dent. Pract..

[55] Ting TY, Wong RWK, Rabie ABM (2011). Analysis of genetic polymorphisms in skeletal Class I crowding. Am. J. Orthod. Dentofacial Orthop..

[56] Singh B (2020). Evaluation of normal morphology of mandibular condyle: a radiographic survey. J. Clin. Imaging Sci..

[57] Little J (2009). Strengthening the reporting of genetic association studies (STREGA): An extension of the strengthening the reporting of observational studies in epidemiology (STROBE) statement. J. Clin. Epidemiol..

[58] Godinho NM (2008). Regional patterns of genetic admixture in south America. Forensic Sci. Int. Genet. Suppl. Ser..

[59] Kuc-Michalska M, Baccetti T (2010). Duration of the pubertal peak in skeletal Class I and Class III subjects. Angle Orthod..

[60] Ruellas ACO (2016). Comparison and reproducibility of 2 regions of reference for maxillary regional registration with cone-beam computed tomography. Am. J. Orthod. Dentofacial Orthop..

[61] Mardia KV, Bookstein FL, Moreton IJ (2000). Statistical assessment of bilateral symmetry of shapes. Biometrika.

[62] Klingenberg CP, Barluenga M, Meyer A (2002). Shape analysis of symmetric structures: Quantifying variation among individuals and asymmetry. Evolution.

[63] Küchler EC (2012). Buccal cells DNA extraction to obtain high quality human genomic DNA suitable for polymorphism genotyping by PCR-RFLP and Real-Time PCR. J. Appl. Oral Sci..

[64] Ranade K (2001). High-throughput genotyping with single nucleotide polymorphisms. Genome Res..

