# Supplementary material for: BMP2 rs1005464 is associated with mandibular condyle size variation
Source: Sci Rep. 2024 Mar 12;14:5987. doi: 10.1038/s41598-024-56530-3 (PMC10933287; doi:10.1038/s41598-024-56530-3)
Supplement: Supplementary file 2 — Supplementary Tables. [file 41598_2024_56530_MOESM2_ESM.pdf]

Supplementary Table S1. 3D landmarks identification method error.

Landmark	Coordinate	ICC (95% CI)	Dahlberg's Formula	Bland-Altman Bias - Estimate (95% CI)
SCr	X	0.99 (0.96, 0.99)	0.22	-0.11 (-0.24, 0.02)
	Y	1.00 (0.99, 1.00)	0.17	-0.03 (-0.14, 0.07)
	Z	1.00 (1.00, 1.00)	0.13	-0.06 (-0.13, 0.02)
PCr	X	0.97 (0.93, 0.99)	0.41	-0.11 (-0.35, 0.13)
	Y	0.99 (0.98, 1.00)	0.33	-0.13 (-0.32, 0.07)
	Z	1.00 (1.00, 1.00)	0.15	0.04 (-0.06, 0.13)
LPr	X	0.94 (0.87, 0.97)	0.37	0.00 (-0.22, 0.23)
	Y	0.99 (0.98, 1.00)	0.37	-0.13 (-0.34, 0.09)
	Z	0.99 0.98, 1.00)	0.44	0.05 (-0.21, 0.32)
MPr	X	0.96 (0.91, 0.98)	0.25	0.04 (-0.12, 0.20)
	Y	1.00 (0.99, 1.00)	0.24	0.06 (-0.09, 0.20)
	Z	1.00 (0.99, 1.00)	0.24	-0.11 (-0.25, 0.03)
SNr	X	0.99 (0.98, 1.00)	0.20	-0.03 (-0.15, 0.09)
	Y	0.99 (0.98, 1.00)	0.31	0.13 (-0.05, 0.30)
	Z	1.00 (1.00, 1.00)	0.19	-0.07 (-0.18, 0.04)
CPr	X	0.99 (0.99, 1.00)	0.21	-0.10 (-0.22, 0.01)
	Y	1.00 0.99, 1.00)	0.27	0.06 (-0.10, 0.22)
	Z	1.00 (1.00, 1.00)	0.13	-0.05 (-0.12, 0.03)
SCl	X	0.91 (0.79, 0.96)	0.66	0.25 (-0.18, 0.68)
	Y	0.99 (0.97, 1.00)	0.32	-0.09 (-0.31, 0.13)
	Z	1.00 (0.99, 1.00)	0.17	-0.04 (-0.16, 0.07)
PCl	X	0.94 (0.86, 0.97)	0.44	-0.13 (-0.40, 0.15)
	Y	1.00 (0.99, 1.00)	0.23	0.10 (-0.04, 0.24)
	Z	0.99 (0.98, 1.00)	0.29	0.12 (-0.07, 0.30)
LPl	X	0.93 (0.84, 0.97)	0.58	-0.02 (-0.40, 0.37)
	Y	0.99 (0.98, 1.00)	0.38	-0.21 (-0.44, 0.02)
	Z	1.00 (0.99, 1.00)	0.17	-0.09 (-0.20, 0.02)
MPl	X	0.93 (0.83, 0.97)	0.42	-0.19 (-0.43, 0.06)
	Y	1.00 (0.99, 1.00)	0.27	0.14 (-0.01, 0.30)
	Z	1.00 (0.99, 1.00)	0.28	-0.09 (-0.26, 0.08)
SNl	X	0.99 (0.98, 1.00)	0.19	0.02 (-0.10, 0.14)
	Y	1.00 (0.99, 1.00)	0.37	0.19 (-0.02, 0.40)
	Z	1.00 (1.00, 1.00)	0.21	-0.11 (-0.23, 0.02)
CPl	X	0.99 (0.97, 0.99)	0.34	0.07 (-0.14, 0.28)
	Y	0.99 (0.98, 1.00)	0.46	0.16 (-0.12, 0.43)
	Z	1.00 (1.00, 1.00)	0.17	0.01 (-0.09, 0.12)

ICC – intraclass correlation coefficient, CI – confidence interval, SCr – right superior Condilion, PCr – right posterior Condilion, LPr – right lateral pole, MPr – right medial pole, SNr – right Sigmoid notch, CPr – right Coronoid process, SCl – left superior Condilion, PCl – left posterior Condilion, LPl – left lateral pole, MPl – left medial pole, SNl – left Sigmoid notch, CPl – left Coronoid process.

Supplementary Table S2. Volume measurement method error.

Measurement	ICC (95% CI)	Dahlberg's Formula	Bland-Altman Bias - Estimate (95% CI)
Right condyle vol.	0.99 (0.98, 1.00)	51.89	-22.20 (-52.30, 8.00)
Left condyle vol.	1.00 (0.99, 1.00)	36.77	-16.30 (-37.60, 5.00)

ICC – intraclass correlation coefficient, CI – confidence interval, vol. – volume.

Supplementary Table S3. Description of mandibular condyles shape configurations for most negative (-β) or positive (+β) PC scores.

PCs	Symmetric component		Asymmetric component	
	**-β**	**+β**	**-β**	**+β**
PC1	Condyles anteroposteriorly and vertically larger; increased SCo - PCo distance; LPs showing outward yaw and upward roll; MPs showing downward roll.	Condyles anteroposteriorly and vertically smaller; decreased SCo - PCo distance; LPs showing inward yaw and downward roll; MPs showing upward roll.	Left condyle vertically and anteroposteriorly larger than the right condyle; left condyle showing clockwise pitch and counterclockwise roll; right condyle showing counterclockwise roll, slight counterclockwise pitch and SCo located more laterally.	Right condyle vertically and anteroposteriorly larger than the left condyle; right condyle showing clockwise pitch and roll; left condyle showing clockwise roll, slight counterclockwise pitch and SCo located more laterally.
PC2	Condyles anteroposteriorly larger; SCos located more posteriorly; MPs showing inward yaw and upward roll.	Condyles anteroposteriorly smaller; SCo located more anteriorly; MPs showing outward yaw and downward roll.	Left condyle vertically and anteroposteriorly larger than the right condyle; left condyle showing counterclockwise yaw and MP with upward roll; right condyle showing counterclockwise yaw and MP with downward roll.	Right condyle vertically and anteroposteriorly larger than the left condyle; right condyle showing clockwise yaw and MP with upward roll; left condyle showing clockwise yaw and MP with downward roll.
PC3	Head condyles transversely and vertically larger; right and left condyles showing markedly counterclockwise and clockwise roll, respectively.	Head condyles transversely and vertically smaller; right and left condyles showing markedly clockwise and counterclockwise roll, respectively.	Left condyle anteroposteriorly and vertically larger than the right condyle; left condyle showing counterclockwise roll, MP with inward yaw and decreased SCo - PCo distance; right condyle showing increased SCo - PCo distance, counterclockwise roll, and MP with slight outward yaw.	Right condyle anteroposteriorly and vertically larger than the left condyle; right condyle showing clockwise roll, MP with inward yaw and decreased SCo - PCo distance; left condyle showing increased SCo - PCo distance, clockwise roll, and MP with slight outward yaw.
PC4	Head condyles transversely larger and anteroposteriorly narrower; SCos located more laterally; PCos located more medially; LPs showing upward roll.	Head condyles transversely narrower and anteroposteriorly larger; SCos located more medially; PCos located more laterally; LPs showing downward roll.	Left condyle anteroposteriorly shorter than the left condyle; left head condyle transversely shorter than the right head condyle; left condyle showing counterclockwise yaw, MP with upward roll and PCo located more anteriorly and laterally; right condyle showing counterclockwise yaw, MP with downward roll and PCo located more posteriorly and medially.	Right condyle anteroposteriorly shorter than the left condyle; right head condyle transversely shorter than the left head condyle; right condyle showing clockwise yaw, MP with upward roll and PCo located more anteriorly and laterally; left condyle showing clockwise yaw, MP with downward roll and PCo located more posteriorly and medially.
PC5	Condyles vertically larger and anteroposteriorly smaller; SCos located more anteriorly and laterally; PCos located more inferiorly; LPs showing upward roll.	Condyles vertically shorter and anteroposteriorly larger; SCos located more posteriorly and medially; PCos located more superiorly; LPs showing downward roll.	Left condyle vertically and anteroposteriorly larger than the right condyle; left condyle with increased SCo - PCo distance, LP with outward yaw and upward roll; right condyle with decreased SCo - PCo distance, LP with inward yaw and downward roll.	Right condyle vertically and anteroposteriorly larger than the left condyle; right condyle with increased SCo - PCo distance, LP with outward yaw and upward roll; left condyle with decreased SCo - PCo distance, LP with inward yaw and downward roll.
PC6	Head condyles transversally shorter and showing outward yaw and counterclockwise pitch; SCos located more medially; PCos located more laterally; MPs showing upward roll.	Head condyles transversally larger and showing inward yaw and clockwise pitch; SCos located more laterally; PCos located more medially; MPs showing downward roll.	Left condyle vertically larger than the right condyle; left condyle head transversely larger than the right condyle head; left condyle showing clockwise yaw and roll, and SCo and PCo located more medially; left condyle showing clockwise yaw and roll, and SCo and PCo located more laterally.	Right condyle vertically larger than the left condyle; right condyle head transversely larger than the left condyle head; right condyle showing counterclockwise yaw and roll, and SCo and PCo located more medially; left condyle showing counterclockwise yaw and roll, and SCo and PCo located more laterally.

Supplementary Table S4. Effect of single nucleotide polymorphisms studied on mandibular condyles traits.

Mandibular condyles trait	Gene SNP (1/2)†	Genotypes	Model coefficients				Model fit measures	
			β	95% CI lower	95% CI upper	*P* value	F test *P* value	Adjusted R^2
PC1 - Symmetric component	*BMP2* rs1005464 (A/G)	AG vs. GG	-0.004	-0.017	0.010	0.592	0.959	-0.031
		AA vs. GG	-0.002	-0.028	0.024	0.874		
		AG+AA vs. GG	-0.003	-0.016	0.009	0.597	0.891	-0.022
		AA vs. AG+GG	0.000	-0.026	0.024	0.954	0.952	-0.025
	BMP2 rs235768 (A/T)	AT vs. TT	0.015	-0.011	0.041	0.245	0.789	-0.021
		AA vs. TT	0.002	-0.011	0.015	0.780		
		AT+AA vs. TT	0.003	-0.009	0.016	0.586	0.887	-0.022
		AA vs. AT+TT	0.014	-0.011	0.039	0.256	0.650	-0.012
	BMP4 rs17563 (G/A)	AG vs. AA	-0.006	-0.020	0.008	0.405	0.711	-0.017
		GG vs. AA	0.005	-0.015	0.026	0.612		
		AG+GG vs. AA	-0.004	-0.018	0.010	0.563	0.878	-0.021
		GG vs. AG+AA	0.009	-0.008	0.027	0.297	0.696	-0.014
	RUNX2 rs59983488 (T/G)	GT vs. GG	0.000	-0.013	0.013	0.998	0.883	-0.026
		TT vs. GG	0.017	-0.021	0.056	0.370		
		GT+TT vs. GG	0.001	-0.011	0.014	0.859	0.946	-0.024
		TT vs. GT+GG	0.017	-0.020	0.055	0.364	0.760	-0.017
	RUNX2 rs1200425 (A/G)	AG vs. GG	-0.002	-0.016	0.013	0.829	0.983	-0.034
		AA vs. GG	-0.002	-0.019	0.016	0.850		
		AG+AA vs. GG	-0.002	-0.015	0.012	0.815	0.941	-0.024
		AA vs. AG+GG	0.000	-0.015	0.014	0.928	0.951	-0.025
	SMAD6 rs2119261 (T/C)	CT vs. CC	-0.007	-0.021	0.006	0.281	0.669	-0.015
		TT vs. CC	0.004	-0.015	0.023	0.690		
		CT+TT vs. CC	-0.005	-0.017	0.008	0.458	0.826	-0.019
		TT vs. CT+CC	0.008	-0.009	0.025	0.358	0.755	-0.017
	SMAD6 rs3934908 (T/C)	CT vs. CC	0.000	-0.015	0.013	0.893	0.909	-0.028
		TT vs. CC	0.006	-0.013	0.025	0.517		
		CT+TT vs. CC	0.000	-0.012	0.014	0.910	0.950	-0.024

		TT vs. CT+CC	0.007	-0.010	0.023	0.421	0.803	-0.018
PC2 - Symmetric component	*BMP2* rs1005464 (A/G)	AG vs. GG	-0.011	-0.021	-0.002	0.020*	0.007*	0.090
		AA vs. GG	-0.011	-0.029	0.007	0.228		
		AG+AA vs. GG	-0.011	-0.020	-0.002	0.014*	0.003*	0.098
		AA vs. AG+GG	-0.007	-0.025	0.011	0.449	0.035*	0.051
	BMP2 rs235768 (A/T)	AT vs. TT	-0.013	-0.032	0.006	0.165	0.039*	0.055
		AA vs. TT	-0.003	-0.012	0.007	0.501		
		AT+AA vs. TT	-0.004	-0.013	0.005	0.338	0.030*	0.054
		AA vs. AT+TT	-0.012	-0.030	0.006	0.206	0.022*	0.060
	BMP4 rs17563 (G/A)	AG vs. AA	0.000	-0.010	0.011	0.902	0.081	0.040
		GG vs. AA	-0.003	-0.018	0.012	0.676		
		AG+GG vs. AA	0.000	-0.010	0.010	0.999	0.045*	0.046
		GG vs. AG+AA	-0.004	-0.017	0.009	0.583	0.040*	0.048
	RUNX2 rs59983488 (T/G)	GT vs. GG	-0.005	-0.014	0.005	0.314	0.046*	0.052
		TT vs. GG	0.009	-0.018	0.037	0.503		
		GT+TT vs. GG	-0.004	-0.013	0.005	0.409	0.033*	0.052
		TT vs. GT+GG	0.011	-0.016	0.039	0.418	0.034*	0.051
	RUNX2 rs1200425 (A/G)	AG vs. GG	0.000	-0.011	0.010	0.870	0.058	0.047
		AA vs. GG	0.005	-0.007	0.018	0.422		
		AG+AA vs. GG	0.000	-0.009	0.011	0.848	0.045*	0.046
		AA vs. AG+GG	0.006	-0.005	0.016	0.294	0.027*	0.055
	SMAD6 rs2119261 (T/C)	CT vs. CC	0.003	-0.007	0.012	0.584	0.081	0.039
		TT vs. CC	0.001	-0.012	0.015	0.835		
		CT+TT vs. CC	0.002	-0.007	0.012	0.604	0.040*	0.048
		TT vs. CT+CC	0.000	-0.013	0.012	0.986	0.045*	0.046
	SMAD6 rs3934908 (T/C)	CT vs. CC	-0.006	-0.016	0.004	0.228	0.038*	0.055
		TT vs. CC	0.001	-0.012	0.014	0.876		
		CT+TT vs. CC	-0.004	-0.014	0.005	0.364	0.031*	0.053
		TT vs. CT+CC	0.005	-0.007	0.017	0.420	0.034*	0.051
PC3 - Symmetric	*BMP2* rs1005464 (A/G)	AG vs. GG	0.000	-0.008	0.008	0.982	0.404	0.000
		AA vs. GG	-0.007	-0.022	0.008	0.358		

component		AG+AA vs. GG	-0.001	-0.008	0.006	0.784	0.361	0.002
		AA vs. AG+GG	-0.007	-0.022	0.008	0.345	0.258	0.010
	BMP2 rs235768 (A/T)	AT vs. TT	0.003	-0.012	0.018	0.663	0.223	0.016
		AA vs. TT	-0.005	-0.013	0.002	0.167		
		AT+AA vs. TT	-0.004	-0.011	0.003	0.253	0.217	0.014
		AA vs. AT+TT	0.006	-0.009	0.021	0.420	0.285	0.007
	BMP4 rs17563 (G/A)	AG vs. AA	-0.002	-0.010	0.006	0.595	0.164	0.023
		GG vs. AA	0.008	-0.004	0.020	0.200		
		AG+GG vs. AA	0.000	-0.009	0.008	0.901	0.370	0.002
		GG vs. AG+AA	0.009	-0.001	0.020	0.078	0.100	0.030
	RUNX2 rs59983488 (T/G)	GT vs. GG	0.002	-0.006	0.010	0.601	0.494	-0.005
		TT vs. GG	0.000	-0.022	0.023	0.950		
		GT+TT vs. GG	0.002	-0.005	0.009	0.607	0.334	0.004
		TT vs. GT+GG	0.000	-0.022	0.022	0.995	0.372	0.001
	RUNX2 rs1200425 (A/G)	AG vs. GG	0.000	-0.009	0.008	0.867	0.439	-0.002
		AA vs. GG	0.003	-0.007	0.013	0.564		
		AG+AA vs. GG	0.000	-0.008	0.008	0.920	0.371	0.002
		AA vs. AG+GG	0.003	-0.005	0.012	0.433	0.290	0.007
	SMAD6 rs2119261 (T/C)	CT vs. CC	0.004	-0.004	0.012	0.353	0.392	0.001
		TT vs. CC	0.000	-0.011	0.012	0.939		
		CT+TT vs. CC	0.003	-0.004	0.010	0.433	0.290	0.007
		TT vs. CT+CC	-0.002	-0.012	0.008	0.738	0.356	0.002
	SMAD6 rs3934908 (T/C)	CT vs. CC	0.003	-0.005	0.011	0.430	0.423	0.000
		TT vs. CC	0.004	-0.007	0.015	0.494		
		CT+TT vs. CC	0.003	-0.004	0.011	0.387	0.274	0.008
		TT vs. CT+CC	0.002	-0.008	0.011	0.719	0.353	0.003
PC4 - Symmetric component	*BMP2* rs1005464 (A/G)	AG vs. GG	0.005	-0.001	0.012	0.120	0.159	0.024
		AA vs. GG	0.008	-0.005	0.020	0.241		
		AG+AA vs. GG	0.006	0.000	0.012	0.079	0.090	0.032
		AA vs. AG+GG	0.006	-0.007	0.018	0.376	0.244	0.011
	BMP2 rs235768 (A/T)	AT vs. TT	0.002	-0.011	0.015	0.719	0.449	-0.002

		AA vs. TT	-0.001	-0.008	0.005	0.728		
		AT+AA vs. TT	0.000	-0.007	0.006	0.821	0.331	0.004
		AA vs. AT+TT	0.003	-0.010	0.016	0.643	0.309	0.006
	BMP4 rs17563 (G/A)	AG vs. AA	-0.005	-0.012	0.002	0.193	0.250	0.013
		GG vs. AA	0.000	-0.011	0.010	0.885		
		AG+GG vs. AA	-0.004	-0.011	0.003	0.251	0.195	0.016
		GG vs. AG+AA	0.003	-0.007	0.012	0.575	0.297	0.007
	RUNX2 rs59983488 (T/G)	GT vs. GG	0.003	-0.003	0.010	0.308	0.235	0.015
		TT vs. GG	-0.009	-0.028	0.010	0.356		
		GT+TT vs. GG	0.003	-0.004	0.009	0.432	0.262	0.009
		TT vs. GT+GG	-0.010	-0.029	0.009	0.286	0.210	0.014
	RUNX2 rs1200425 (A/G)	AG vs. GG	0.000	-0.007	0.007	0.954	0.347	0.005
		AA vs. GG	0.004	-0.005	0.013	0.358		
		AG+AA vs. GG	0.001	-0.005	0.008	0.689	0.316	0.005
		AA vs. AG+GG	0.004	-0.003	0.011	0.294	0.214	0.014
	SMAD6 rs2119261 (T/C)	CT vs. CC	-0.002	-0.009	0.005	0.614	0.459	-0.003
		TT vs. CC	0.000	-0.010	0.009	0.897		
		CT+TT vs. CC	-0.001	-0.008	0.005	0.648	0.310	0.006
		TT vs. CT+CC	0.000	-0.008	0.009	0.933	0.337	0.004
	SMAD6 rs3934908 (T/C)	CT vs. CC	-0.003	-0.010	0.004	0.466	0.399	0.000
		TT vs. CC	-0.003	-0.013	0.006	0.493		
		CT+TT vs. CC	-0.003	-0.009	0.004	0.414	0.256	0.010
		TT vs. CT+CC	-0.002	-0.010	0.007	0.694	0.317	0.005
PC5 - Symmetric component	*BMP2* rs1005464 (A/G)	AG vs. GG	-0.004	-0.010	0.001	0.127	0.009*	0.085
		AA vs. GG	0.000	0.012	0.012	0.885		
		AG+AA vs. GG	-0.004	-0.009	0.002	0.189	0.005*	0.086
		AA vs. AG+GG	0.002	-0.008	0.013	0.658	0.011*	0.073
	BMP2 rs235768 (A/T)	AT vs. TT	0.006	-0.005	0.016	0.314	0.012*	0.080
		AA vs. TT	0.003	-0.002	0.009	0.235		
		AT+AA vs. TT	0.004	-0.002	0.009	0.182	0.005*	0.087
		AA vs. AT+TT	0.004	-0.007	0.015	0.465	0.009*	0.076

	BMP4 rs17563 (G/A)	AG vs. AA	0.001	-0.005	0.007	0.644	0.025*	0.065
		GG vs. AA	0.000	-0.008	0.010	0.831		
		AG+GG vs. AA	0.001	-0.005	0.007	0.653	0.011*	0.073
		GG vs. AG+AA	0.000	-0.008	0.008	0.989	0.012*	0.071
	RUNX2 rs59983488 (T/G)	GT vs. GG	0.000	-0.006	0.005	0.829	0.025*	0.064
		TT vs. GG	0.003	-0.014	0.019	0.758		
		GT+TT vs. GG	0.000	-0.006	0.005	0.885	0.012*	0.072
		TT vs. GT+GG	0.003	-0.013	0.019	0.733	0.011*	0.072
	RUNX2 rs1200425 (A/G)	AG vs. GG	0.004	-0.002	0.010	0.156	0.010*	0.082
		AA vs. GG	0.001	-0.006	0.009	0.721		
		AG+AA vs. GG	0.003	-0.002	0.009	0.234	0.006*	0.084
		AA vs. AG+GG	-0.001	-0.008	0.005	0.646	0.011*	0.073
	SMAD6 rs2119261 (T/C)	CT vs. CC	0.004	-0.002	0.009	0.216	0.014*	0.077
		TT vs. CC	0.003	-0.005	0.011	0.463		
		CT+TT vs. CC	0.003	-0.002	0.009	0.208	0.006*	0.085
		TT vs. CT+CC	0.000	-0.006	0.008	0.804	0.011*	0.072
	SMAD6 rs3934908 (T/C)	CT vs. CC	0.000	-0.006	0.050	0.878	0.027*	0.063
		TT vs. CC	0.000	-0.008	0.008	0.987		
		CT+TT vs. CC	0.000	-0.006	0.005	0.907	0.012*	0.072
		TT vs. CT+CC	0.000	-0.007	0.007	0.921	0.012*	0.071
PC6 - Symmetric component	*BMP2* rs1005464 (A/G)	AG vs. GG	0.000	-0.004	0.006	0.733	0.065	0.044
		AA vs. GG	0.007	-0.002	0.017	0.140		
		AG+AA vs. GG	0.002	-0.003	0.007	0.439	0.064	0.039
		AA vs. AG+GG	0.007	-0.003	0.016	0.149	0.032*	0.052
	BMP2 rs235768 (A/T)	AT vs. TT	0.004	-0.006	0.013	0.472	0.068	0.043
		AA vs. TT	-0.003	-0.007	0.002	0.304		
		AT+AA vs. TT	-0.002	-0.007	0.003	0.453	0.065	0.039
		AA vs. AT+TT	0.005	-0.005	0.014	0.313	0.053	0.043
	BMP4 rs17563 (G/A)	AG vs. AA	0.000	-0.005	0.006	0.893	0.127	0.029
		GG vs. AA	-0.002	-0.010	0.006	0.580		
		AG+GG vs. AA	0.000	-0.005	0.005	0.979	0.083	0.034

		GG vs. AG+AA	-0.002	-0.009	0.004	0.477	0.066	0.038
	RUNX2 rs59983488 (T/G)	GT vs. GG	0.003	-0.002	0.007	0.298	0.100	0.035
		TT vs. GG	0.000	-0.015	0.014	0.936		
		GT+TT vs. GG	0.002	-0.002	0.007	0.327	0.054	0.042
		TT vs. GT+GG	-0.002	-0.016	0.013	0.826	0.081	0.034
	RUNX2 rs1200425 (A/G)	AG vs. GG	0.000	-0.005	0.006	0.761	0.136	0.028
		AA vs. GG	0.002	-0.005	0.009	0.551		
		AG+AA vs. GG	0.001	-0.004	0.006	0.647	0.076	0.035
		AA vs. AG+GG	0.001	-0.004	0.007	0.606	0.074	0.036
	SMAD6 rs2119261 (T/C)	CT vs. CC	-0.001	-0.006	0.004	0.629	0.057	0.047
		TT vs. CC	-0.006	-0.013	0.001	0.117		
		CT+TT vs. CC	-0.002	-0.007	0.003	0.358	0.057	0.041
		TT vs. CT+CC	-0.005	-0.012	0.002	0.132	0.030*	0.054
	SMAD6 rs3934908 (T/C)	CT vs. CC	0.002	-0.003	0.008	0.361	0.074	0.041
		TT vs. CC	-0.002	-0.009	0.005	0.636		
		CT+TT vs. CC	0.001	-0.004	0.006	0.571	0.072	0.036
		TT vs. CT+CC	-0.003	-0.009	0.003	0.311	0.052	0.043
PC1 - Asymmetric component	*BMP2* rs1005464 (A/G)	AG vs. GG	0.002	-0.003	0.007	0.504	0.663	-0.015
		AA vs. GG	-0.003	-0.012	0.007	0.615		
		AG+AA vs. GG	0.001	-0.004	0.006	0.665	0.632	-0.012
		AA vs. AG+GG	-0.003	-0.013	0.006	0.519	0.582	-0.009
	BMP2 rs235768 (A/T)	AT vs. TT	0.007	-0.003	0.017	0.180	0.408	0.000
		AA vs. TT	-0.001	-0.006	0.004	0.665		
		AT+AA vs. TT	0.000	-0.005	0.005	0.956	0.674	-0.013
		AA vs. AT+TT	0.007	-0.002	0.017	0.133	0.283	0.008
	BMP4 rs17563 (G/A)	AG vs. AA	-0.004	-0.010	0.001	0.128	0.418	0.000
		GG vs. AA	-0.004	-0.011	0.004	0.372		
		AG+GG vs. AA	-0.004	-0.009	0.001	0.126	0.273	0.008
		GG vs. AG+AA	0.000	-0.008	0.006	0.866	0.668	-0.013
	RUNX2 rs59983488 (T/G)	GT vs. GG	-0.003	-0.008	0.002	0.266	0.444	-0.002
		TT vs. GG	-0.008	-0.023	0.006	0.267		

		GT+TT vs. GG	-0.003	-0.008	0.002	0.199	0.362	0.002
		TT vs. GT+GG	-0.007	-0.022	0.007	0.331	0.477	-0.004
	RUNX2 rs1200425 (A/G)	AG vs. GG	-0.001	-0.007	0.004	0.643	0.755	-0.019
		AA vs. GG	0.000	-0.006	0.007	0.933		
		AG+AA vs. GG	0.000	-0.006	0.004	0.756	0.652	-0.012
		AA vs. AG+GG	0.001	-0.005	0.007	0.698	0.640	-0.012
	SMAD6 rs2119261 (T/C)	CT vs. CC	-0.002	-0.007	0.003	0.393	0.406	0.000
		TT vs. CC	0.003	-0.004	0.010	0.396		
		CT+TT vs. CC	-0.001	-0.006	0.004	0.684	0.637	-0.012
		TT vs. CT+CC	0.004	-0.002	0.011	0.189	0.351	0.003
	SMAD6 rs3934908 (T/C)	CT vs. CC	-0.003	-0.008	0.003	0.315	0.600	-0.011
		TT vs. CC	-0.003	-0.010	0.004	0.389		
		CT+TT vs. CC	-0.003	-0.008	0.002	0.273	0.432	-0.002
		TT vs. CT+CC	-0.001	-0.008	0.005	0.655	0.629	-0.012
PC2 - Asymmetric component	*BMP2* rs1005464 (A/G)	AG vs. GG	-0.001	-0.005	0.003	0.497	0.299	0.009
		AA vs. GG	0.003	-0.005	0.010	0.501		
		AG+AA vs. GG	0.000	-0.004	0.003	0.697	0.270	0.009
		AA vs. AG+GG	0.003	-0.004	0.010	0.413	0.217	0.014
	BMP2 rs235768 (A/T)	AT vs. TT	-0.004	-0.012	0.003	0.248	0.232	0.015
		AA vs. TT	-0.002	-0.006	0.002	0.332		
		AT+AA vs. TT	-0.002	-0.006	0.001	0.242	0.161	0.020
		AA vs. AT+TT	-0.003	-0.011	0.004	0.346	0.198	0.016
	BMP4 rs17563 (G/A)	AG vs. AA	0.002	-0.002	0.007	0.229	0.156	0.025
		GG vs. AA	0.005	-0.001	0.011	0.104		
		AG+GG vs. AA	0.003	-0.001	0.007	0.150	0.119	0.026
		GG vs. AG+AA	0.003	-0.002	0.008	0.232	0.157	0.020
	RUNX2 rs59983488 (T/G)	GT vs. GG	0.002	-0.002	0.006	0.330	0.316	0.007
		TT vs. GG	0.000	-0.011	0.011	0.991		
		GT+TT vs. GG	0.002	-0.002	0.005	0.352	0.200	0.015
		TT vs. GT+GG	0.000	-0.012	0.010	0.887	0.286	0.007
	RUNX2 rs1200425 (A/G)	AG vs. GG	0.000	-0.004	0.004	0.934	0.389	0.001
		AA vs. GG	-0.001	-0.006	0.004	0.638		
		AG+AA vs. GG	0.000	-0.004	0.004	0.902	0.286	0.007

		AA vs. AG+GG	-0.001	-0.006	0.003	0.544	0.247	0.011
	SMAD6 rs2119261 (T/C)	CT vs. CC	0.001	-0.003	0.005	0.524	0.179	0.021
		TT vs. CC	-0.003	-0.008	0.003	0.286		
		CT+TT vs. CC	0.000	-0.003	0.004	0.875	0.285	0.008
		TT vs. CT+CC	-0.004	-0.009	0.001	0.146	0.117	0.027
	SMAD6 rs3934908 (T/C)	CT vs. CC	0.000	-0.004	0.004	0.985	0.221	0.016
		TT vs. CC	0.003	-0.002	0.009	0.213		
		CT+TT vs. CC	0.000	-0.003	0.005	0.662	0.266	0.009
		TT vs. CT+CC	0.003	-0.001	0.008	0.162	0.125	0.025
PC3 - Asymmetric component	*BMP2* rs1005464 (A/G)	AG vs. GG	0.000	-0.003	0.004	0.717	0.446	-0.002
		AA vs. GG	0.006	0.000	0.012	0.085		
		AG+AA vs. GG	0.001	-0.002	0.005	0.384	0.691	-0.014
		AA vs. AG+GG	0.005	0.000	0.012	0.090	0.309	0.006
	BMP2 rs235768 (A/T)	AT vs. TT	0.000	-0.007	0.006	0.769	0.658	-0.014
		AA vs. TT	0.002	-0.001	0.005	0.247		
		AT+AA vs. TT	0.002	-0.002	0.005	0.328	0.645	-0.012
		AA vs. AT+TT	-0.002	-0.008	0.004	0.545	0.784	-0.018
	BMP4 rs17563 (G/A)	AG vs. AA	0.002	-0.002	0.005	0.401	0.771	-0.020
		GG vs. AA	0.000	-0.006	0.005	0.891		
		AG+GG vs. AA	0.001	-0.002	0.005	0.495	0.760	-0.017
		GG vs. AG+AA	-0.001	-0.006	0.003	0.531	0.778	-0.018
	RUNX2 rs59983488 (T/G)	GT vs. GG	0.000	-0.004	0.005	0.722	0.935	-0.030
		TT vs. GG	0.000	-0.010	0.009	0.958		
		GT+TT vs. GG	0.000	-0.004	0.003	0.725	0.843	-0.020
		TT vs. GT+GG	0.000	-0.010	0.010	0.996	0.873	-0.021
	RUNX2 rs1200425 (A/G)	AG vs. GG	0.002	-0.002	0.005	0.373	0.201	0.019
		AA vs. GG	-0.003	-0.007	0.001	0.181		
		AG+AA vs. GG	0.000	-0.003	0.004	0.893	0.869	-0.021
		AA vs. AG+GG	-0.004	-0.008	0.000	0.035*	0.158	0.020
	SMAD6 rs2119261 (T/C)	CT vs. CC	0.000	-0.003	0.003	0.985	0.659	-0.014
		TT vs. CC	-0.003	-0.008	0.002	0.231		
		CT+TT vs. CC	0.000	-0.004	0.003	0.675	0.831	-0.020
		TT vs. CT+CC	-0.003	-0.007	0.001	0.190	0.488	-0.005
	SMAD6 rs3934908 (T/C)	CT vs. CC	0.000	-0.003	0.004	0.632	0.742	-0.019
		TT vs. CC	-0.002	-0.006	0.003	0.499		

		CT+TT vs. CC	0.000	-0.003	0.004	0.879	0.868	-0.021
		TT vs. CT+CC	-0.002	-0.006	0.002	0.310	0.628	-0.011
PC4 - Asymmetric component	*BMP2* rs1005464 (A/G)	AG vs. GG	-0.002	-0.005	0.001	0.298	0.106	0.033
		AA vs. GG	0.002	-0.004	0.008	0.455		
		AG+AA vs. GG	-0.001	-0.004	0.002	0.493	0.107	0.028
		AA vs. AG+GG	0.003	-0.003	0.009	0.335	0.088	0.033
	BMP2 rs235768 (A/T)	AT vs. TT	0.004	-0.002	0.010	0.215	0.085	0.038
		AA vs. TT	-0.001	-0.004	0.002	0.499		
		AT+AA vs. TT	0.000	-0.003	0.002	0.765	0.127	0.025
		AA vs. AT+TT	0.004	-0.002	0.010	0.144	0.051	0.043
	BMP4 rs17563 (G/A)	AG vs. AA	0.000	-0.003	0.004	0.869	0.193	0.019
		GG vs. AA	-0.001	-0.006	0.004	0.609		
		AG+GG vs. AA	0.000	-0.003	0.003	0.992	0.132	0.024
		GG vs. AG+AA	-0.001	-0.006	0.003	0.496	0.108	0.028
	RUNX2 rs59983488 (T/G)	GT vs. GG	0.002	0.000	0.005	0.132	0.043*	0.053
		TT vs. GG	-0.005	-0.014	0.003	0.230		
		GT+TT vs. GG	0.002	-0.001	0.005	0.235	0.071	0.037
		TT vs. GT+GG	-0.006	-0.015	0.003	0.161	0.055	0.042
	RUNX2 rs1200425 (A/G)	AG vs. GG	0.000	-0.003	0.004	0.675	0.217	0.017
		AA vs. GG	0.000	-0.004	0.005	0.853		
		AG+AA vs. GG	0.000	-0.003	0.004	0.701	0.124	0.025
		AA vs. AG+GG	0.000	-0.004	0.003	0.966	0.132	0.024
	SMAD6 rs2119261 (T/C)	CT vs. CC	-0.003	-0.006	0.000	0.030*	0.009*	0.084
		TT vs. CC	0.002	-0.003	0.006	0.454		
		CT+TT vs. CC	-0.002	-0.005	0.000	0.137	0.049*	0.044
		TT vs. CT+CC	0.004	0.000	0.008	0.078	0.033*	0.052
	SMAD6 rs3934908 (T/C)	CT vs. CC	0.001	-0.002	0.004	0.517	0.195	0.019
		TT vs. CC	0.001	-0.003	0.005	0.633		
		CT+TT vs. CC	0.001	-0.002	0.004	0.496	0.108	0.028
		TT vs. CT+CC	0.000	-0.003	0.004	0.842	0.130	0.024
PC5 - Asymmetric component	*BMP2* rs1005464 (A/G)	AG vs. GG	0.000	-0.002	0.003	0.844	0.168	0.023
		AA vs. GG	0.005	0.000	0.009	0.041*		
		AG+AA vs. GG	0.000	-0.001	0.003	0.411	0.409	0.000
		AA vs. AG+GG	0.005	0.000	0.009	0.040*	0.092	0.032
	BMP2 rs235768 (A/T)	AT vs. TT	-0.002	-0.007	0.003	0.358	0.303	0.008

		AA vs. TT	-0.002	-0.004	0.000	0.128		
		AT+AA vs. TT	-0.002	-0.004	0.000	0.106	0.184	0.017
		AA vs. AT+TT	-0.001	-0.006	0.003	0.582	0.473	-0.004
	BMP4 rs17563 (G/A)	AG vs. AA	0.000	-0.002	0.003	0.871	0.337	0.005
		GG vs. AA	-0.002	-0.006	0.001	0.213		
		AG+GG vs. AA	0.000	-0.003	0.002	0.858	0.524	-0.007
		GG vs. AG+AA	-0.003	-0.006	0.000	0.128	0.209	0.014
	RUNX2 rs59983488 (T/G)	GT vs. GG	0.000	-0.002	0.002	0.966	0.640	-0.013
		TT vs. GG	-0.002	-0.009	0.005	0.569		
		GT+TT vs. GG	0.000	-0.002	0.002	0.877	0.526	-0.007
		TT vs. GT+GG	-0.002	-0.009	0.005	0.568	0.469	-0.004
	RUNX2 rs1200425 (A/G)	AG vs. GG	-0.001	-0.004	0.001	0.385	0.535	-0.008
		AA vs. GG	-0.001	-0.005	0.002	0.418		
		AG+AA vs. GG	-0.001	-0.004	0.001	0.337	0.371	0.002
		AA vs. AG+GG	0.000	-0.003	0.002	0.678	0.497	-0.005
	SMAD6 rs2119261 (T/C)	CT vs. CC	-0.001	-0.004	0.001	0.376	0.298	0.009
		TT vs. CC	0.002	-0.002	0.005	0.370		
		CT+TT vs. CC	0.000	-0.003	0.002	0.678	0.497	-0.005
		TT vs. CT+CC	0.002	0.000	0.005	0.168	0.248	0.011
	SMAD6 rs3934908 (T/C)	CT vs. CC	0.000	-0.002	0.003	0.868	0.607	-0.012
		TT vs. CC	0.001	-0.002	0.005	0.491		
		CT+TT vs. CC	0.000	-0.002	0.003	0.713	0.504	-0.006
		TT vs. CT+CC	0.001	-0.002	0.004	0.488	0.441	-0.003
PC6 - Asymmetric component	*BMP2* rs1005464 (A/G)	AG vs. GG	-0.003	-0.005	0.000	0.016*	0.064	0.045
		AA vs. GG	0.003	-0.002	0.007	0.231		
		AG+AA vs. GG	-0.002	-0.004	0.000	0.084	0.343	0.003
		AA vs. AG+GG	0.004	0.000	0.008	0.099	0.381	0.000
	BMP2 rs235768 (A/T)	AT vs. TT	0.006	0.001	0.011	0.010*	0.135	0.028
		AA vs. TT	0.000	-0.002	0.003	0.594		
		AT+AA vs. TT	0.001	0.000	0.004	0.266	0.666	-0.013
		AA vs. AT+TT	0.006	0.001	0.010	0.012*	0.080	0.034
	BMP4 rs17563 (G/A)	AG vs. AA	0.000	-0.002	0.003	0.685	0.766	-0.020
		GG vs. AA	-0.002	-0.005	0.002	0.417		
		AG+GG vs. AA	0.000	-0.002	0.003	0.890	0.952	-0.025
		GG vs. AG+AA	-0.002	-0.005	0.001	0.247	0.643	-0.012

	RUNX2 rs59983488 (T/G)	GT vs. GG	0.000	-0.002	0.003	0.815	0.922	-0.029
		TT vs. GG	0.003	-0.004	0.010	0.449		
		GT+TT vs. GG	0.000	-0.002	0.003	0.707	0.927	-0.023
		TT vs. GT+GG	0.003	-0.004	0.009	0.462	0.833	-0.020
	RUNX2 rs1200425 (A/G)	AG vs. GG	0.000	-0.003	0.002	0.801	0.912	-0.028
		AA vs. GG	-0.001	-0.004	0.002	0.431		
		AG+AA vs. GG	0.000	-0.003	0.002	0.621	0.904	-0.022
		AA vs. AG+GG	-0.001	-0.004	0.002	0.439	0.819	-0.019
	SMAD6 rs2119261 (T/C)	CT vs. CC	0.000	-0.003	0.002	0.929	0.974	-0.033
		TT vs. CC	0.000	-0.004	0.003	0.689		
		CT+TT vs. CC	0.000	-0.003	0.002	0.835	0.947	-0.024
		TT vs. CT+CC	0.000	-0.004	0.003	0.689	0.922	-0.023
	SMAD6 rs3934908 (T/C)	CT vs. CC	0.002	0.000	0.004	0.176	0.664	-0.015
		TT vs. CC	0.000	-0.003	0.004	0.825		
		CT+TT vs. CC	0.001	0.000	0.004	0.248	0.644	-0.012
		TT vs. CT+CC	0.000	-0.004	0.002	0.646	0.911	-0.023
Symmetric Log centroid size	*BMP2* rs1005464 (A/G)	AG vs. GG	0.043	0.013	0.073	0.005*	<0.001*	0.165
		AA vs. GG	0.041	-0.017	0.099	0.168		
		AG+AA vs. GG	0.043	0.014	0.071	0.004*	<0.001*	0.173
		AA vs. AG+GG	0.025	-0.034	0.084	0.406	0.001*	0.110
	BMP2 rs235768 (A/T)	AT vs. TT	-0.019	-0.080	0.042	0.535	0.004*	0.101
		AA vs. TT	0.003	-0.033	0.033	0.863		
		AT+AA vs. TT	0.000	-0.029	0.029	0.999	0.002*	0.105
		AA vs. AT+TT	-0.021	-0.079	0.038	0.491	0.001*	0.109
	BMP4 rs17563 (G/A)	AG vs. AA	-0.002	-0.035	0.032	0.921	0.003*	0.107
		GG vs. AA	-0.025	-0.073	0.024	0.315		
		AG+GG vs. AA	-0.006	-0.038	0.027	0.734	0.002*	0.106
		GG vs. AG+AA	-0.024	-0.066	0.019	0.271	0.001*	0.115
	RUNX2 rs59983488 (T/G)	GT vs. GG	0.000	-0.029	0.031	0.956	0.002*	0.111
		TT vs. GG	-0.059	-0.149	0.031	0.196		
		GT+TT vs. GG	-0.003	-0.033	0.026	0.836	0.002*	0.105
		TT vs. GT+GG	-0.059	-0.148	0.029	0.188	<0.001*	0.119
	RUNX2 rs1200425 (A/G)	AG vs. GG	0.004	-0.030	0.038	0.812	0.005*	0.097
		AA vs. GG	0.003	-0.039	0.044	0.895		
		AG+AA vs. GG	0.004	-0.028	0.036	0.819	0.002*	0.105

		AA vs. AG+GG	0.000	-0.035	0.035	0.993	0.002*	0.105
	SMAD6 rs2119261 (T/C)	CT vs. CC	0.004	-0.027	0.036	0.782	0.004*	0.102
		TT vs. CC	-0.013	-0.058	0.032	0.576		
		CT+TT vs. CC	0.000	-0.029	0.031	0.971	0.002*	0.105
		TT vs. CT+CC	-0.015	-0.056	0.026	0.460	0.001*	0.109
	SMAD6 rs3934908 (T/C)	CT vs. CC	0.007	-0.026	0.040	0.671	0.004*	0.101
		TT vs. CC	-0.007	-0.052	0.036	0.728		
		CT+TT vs. CC	0.003	-0.028	0.035	0.825	0.002*	0.105
		TT vs. CT+CC	-0.012	-0.051	0.027	0.538	0.002*	0.108
Asymmetric Log centroid size	*BMP2* rs1005464 (A/G)	AG vs. GG	0.010	-0.007	0.026	0.254	0.360	0.004
		AA vs. GG	0.031	-0.001	0.063	0.058		
		AG+AA vs. GG	0.013	-0.003	0.029	0.107	0.438	-0.002
		AA vs. AG+GG	0.028	-0.004	0.059	0.087	0.384	0.000
	BMP2 rs235768 (A/T)	AT vs. TT	-0.032	-0.065	0.000	0.055	0.402	0.000
		AA vs. TT	0.000	-0.016	0.016	0.971		
		AT+AA vs. TT	-0.004	-0.020	0.012	0.609	0.950	-0.025
		AA vs. AT+TT	-0.032	-0.063	0.000	0.048*	0.256	0.010
	BMP4 rs17563 (G/A)	AG vs. AA	-0.003	-0.021	0.015	0.721	0.994	-0.035
		GG vs. AA	-0.001	-0.028	0.026	0.940		
		AG+GG vs. AA	-0.003	-0.021	0.015	0.746	0.979	-0.026
		GG vs. AG+AA	0.001	-0.022	0.024	0.910	0.992	-0.027
	RUNX2 rs59983488 (T/G)	GT vs. GG	0.003	-0.014	0.019	0.741	0.981	-0.033
		TT vs. GG	0.013	-0.037	0.062	0.612		
		GT+TT vs. GG	0.003	-0.013	0.019	0.675	0.967	-0.088
		TT vs. GT+GG	0.012	-0.037	0.060	0.638	0.958	-0.025
	RUNX2 rs1200425 (A/G)	AG vs. GG	0.015	-0.004	0.033	0.113	0.335	0.006
		AA vs. GG	-0.004	-0.026	0.018	0.721		
		AG+AA vs. GG	0.009	-0.008	0.026	0.306	0.766	-0.017
		AA vs. AG+GG	-0.013	-0.032	0.006	0.165	0.566	-0.009
	SMAD6 rs2119261 (T/C)	CT vs. CC	0.007	-0.010	0.024	0.418	0.658	-0.014
		TT vs. CC	-0.010	-0.035	0.014	0.397		
		CT+TT vs. CC	0.003	-0.013	0.019	0.710	0.973	-0.026
		TT vs. CT+CC	-0.014	-0.036	0.008	0.197	0.622	-0.011
	SMAD6 rs3934908 (T/C)	CT vs. CC	0.007	-0.011	0.025	0.446	0.885	-0.026
		TT vs. CC	0.012	-0.012	0.036	0.334		

		CT+TT vs. CC	0.008	-0.009	0.025	0.348	0.807	-0.019
		TT vs. CT+CC	0.007	-0.014	0.029	0.487	0.902	-0.022
Mahalanobis shape FA scores	*BMP2* rs1005464 (A/G)	AG vs. GG	0.241	-0.131	0.613	0.202	0.011*	0.082
		AA vs. GG	0.917	0.195	1.639	0.013*		
		AG+AA vs. GG	0.347	-0.010	0.704	0.057	0.019*	0.063
		AA vs. AG+GG	0.828	0.117	1.539	0.023*	0.009*	0.076
	BMP2 rs235768 (A/T)	AT vs. TT	0.688	-0.054	1.430	0.069	0.046*	0.051
		AA vs. TT	0.115	-0.252	0.482	0.537		
		AT+AA vs. TT	0.184	-0.175	0.542	0.312	0.062	0.040
		AA vs. AT+TT	0.630	-0.087	1.346	0.084	0.025*	0.057
	BMP4 rs17563 (G/A)	AG vs. AA	0.109	-0.294	0.511	0.594	0.024*	0.065
		GG vs. AA	0.641	0.053	1.228	0.033*		
		AG+GG vs. AA	0.199	-0.199	0.597	0.324	0.063	0.039
		GG vs. AG+AA	0.563	0.052	1.075	0.031*	0.012*	0.071
	RUNX2 rs59983488 (T/G)	GT vs. GG	-0.293	-0.660	0.074	0.117	0.050	0.050
		TT vs. GG	-0.586	-1.686	0.515	0.294		
		GT+TT vs. GG	-0.312	-0.670	0.046	0.087	0.026*	0.056
		TT vs. GT+GG	-0.470	-1.568	0.629	0.399	0.071	0.037
	RUNX2 rs1200425 (A/G)	AG vs. GG	0.133	-0.284	0.550	0.529	0.153	0.025
		AA vs. GG	0.069	-0.438	0.575	0.788		
		AG+AA vs. GG	0.114	-0.279	0.506	0.567	0.084	0.033
		AA vs. AG+GG	-0.016	-0.445	0.414	0.941	0.097	0.030
	SMAD6 rs2119261 (T/C)	CT vs. CC	-0.318	-0.700	0.064	0.102	0.029*	0.061
		TT vs. CC	0.150	-0.393	0.692	0.586		
		CT+TT vs. CC	-0.213	-0.580	0.155	0.254	0.054	0.042
		TT vs. CT+CC	0.334	-0.164	0.833	0.187	0.044*	0.046
	SMAD6 rs3934908 (T/C)	CT vs. CC	-0.107	-0.509	0.295	0.599	0.158	0.024
		TT vs. CC	-0.017	-0.559	0.524	0.949		
		CT+TT vs. CC	-0.086	-0.468	0.296	0.658	0.089	0.032
		TT vs. CT+CC	0.050	-0.429	0.528	0.838	0.095	0.031
Condylar volume (right and left)	*BMP2* rs1005464 (A/G)	AG vs. GG	235.6	43.0	428.2	0.018*	<0.001*	0.191
		AA vs. GG	218.5	-155.4	592.3	0.255		
		AG+AA vs. GG	232.9	50.8	415.0	0.014*	<0.001*	0.192
		AA vs. AG+GG	131.1	-243.8	506.0	0.495	<0.001*	0.154
	BMP2 rs235768 (A/T)	AT vs. TT	106.6	-83.8	297.0	0.275	<0.001*	0.164
		AA vs. TT	-132.5	-517.6	252.7	0.502		

		AT+AA vs. TT	77.9	-107.3	263.0	0.411	<0.001*	0.155
		AA vs. AT+TT	-186.9	-559.9	186.2	0.328	<0.001*	0.157
	BMP4 rs17563 (G/A)	AG vs. AA	-21.1	-230.9	188.7	0.844	<0.001*	0.169
		GG vs. AA	-243.6	-549.7	62.5	0.122		
		AG+GG vs. AA	-58.9	-264.9	147.1	0.576	<0.001*	0.153
		GG vs. AG+AA	-228.6	-494.9	37.6	0.095	<0.001*	0.170
	RUNX2 rs59983488 (T/G)	GT vs. GG	-131.6	-322.0	58.9	0.179	<0.001*	0.162
		TT vs. GG	-65.7	-637.2	505.7	0.822		
		GT+TT vs. GG	-127.3	-313.1	58.6	0.182	<0.001*	0.163
		TT vs. GT+GG	-13.6	-582.2	555.1	0.963	<0.001*	0.150
	RUNX2 rs1200425 (A/G)	AG vs. GG	53.1	-162.1	268.2	0.630	<0.001*	0.154
		AA vs. GG	-38.3	-299.3	222.7	0.774		
		AG+AA vs. GG	25.4	-177.2	228.0	0.806	<0.001*	0.151
		AA vs. AG+GG	-72.2	-293.4	149.0	0.524	<0.001*	0.153
	SMAD6 rs2119261 (T/C)	CT vs. CC	124.0	-75.0	323.0	0.225	<0.001*	0.165
		TT vs. CC	189.5	-93.2	472.2	0.192		
		CT+TT vs. CC	138.8	-49.9	327.5	0.152	<0.001*	0.165
		TT vs. CT+CC	117.4	-141.1	375.9	0.375	<0.001*	0.156
	SMAD6 rs3934908 (T/C)	CT vs. CC	49.6	-157.8	257.1	0.640	<0.001*	0.152
		TT vs. CC	-17.8	-297.1	261.5	0.901		
		CT+TT vs. CC	33.4	-163.8	230.6	0.740	<0.001*	0.151
		TT vs. CT+CC	-48.8	-295.4	197.9	0.699	<0.001*	0.151
Right-left condylar volume difference	*BMP2* rs1005464 (A/G)	AG vs. GG	75.2	-38.7	189.1	0.194	0.383	0.002
		AA vs. GG	116.6	-104.6	337.7	0.298		
		AG+AA vs. GG	81.7	-26.1	189.4	0.136	0.255	0.010
		AA vs. AG+GG	88.7	-129.1	306.4	0.421	0.481	-0.005
	BMP2 rs235768 (A/T)	AT vs. TT	-243.5	-464.7	-22.2	0.031*	0.163	0.024
		AA vs. TT	-39.2	-148.5	70.2	0.479		
		AT+AA vs. TT	-63.7	-171.0	43.5	0.241	0.362	0.002
		AA vs. AT+TT	-223.5	-437.1	-9.9	0.040*	0.109	0.028
	BMP4 rs17563 (G/A)	AG vs. AA	7.6	-115.9	131.1	0.903	0.741	-0.019
		GG vs. AA	-24.9	-205.0	155.3	0.785		
		AG+GG vs. AA	2.1	-117.8	122.0	0.973	0.611	-0.011
		GG vs. AG+AA	-30.2	-186.9	126.4	0.703	0.580	-0.009
	RUNX2 rs59983488 (T/G)	GT vs. GG	-24.1	-135.4	87.2	0.669	0.633	-0.013

	TT vs. GG	116.4	-217.4	450.3	0.491		
	GT+TT vs. GG	-14.9	-123.8	94.0	0.787	0.596	-0.010
	TT vs. GT+GG	126.0	-203.7	455.6	0.450	0.495	-0.005
RUNX2 rs1200425 (A/G)	AG vs. GG	60.4	-64.5	185.3	0.340	0.603	-0.011
	AA vs. GG	42.7	-108.8	194.2	0.578		
	AG+AA vs. GG	55.0	-62.3	172.4	0.354	0.443	-0.003
	AA vs. AG+GG	4.1	-124.7	132.9	0.950	0.611	-0.011
SMAD6 rs2119261 (T/C)	CT vs. CC	-30.3	-147.0	86.4	0.608	0.706	-0.017
	TT vs. CC	4.7	-161.1	170.5	0.955		
	CT+TT vs. CC	-22.4	-133.0	88.3	0.689	0.577	-0.009
	TT vs. CT+CC	22.3	-128.4	173.1	0.770	0.593	-0.010
SMAD6 rs3934908 (T/C)	CT vs. CC	62.5	-57.6	182.5	0.305	0.540	-0.008
	TT vs. CC	73.7	-88.0	235.3	0.368		
	CT+TT vs. CC	65.2	-48.8	179.1	0.260	0.377	0.001
	TT vs. CT+CC	34.7	-108.6	178.0	0.632	0.563	-0.009

SNP – single nucleotide polymorphism, CI – confidence interval, PC – principal component, log – natural logarithm, FA – fluctuating asymmetry.
† (1 = minor allele / 2 = major allele).
* Indicates statistical significance.

Supplementary Table S5. Single effect of the variables sex, age and malocclusion on mandibular condyles traits.

Mandibular condyles trait	Univariate statistics estimates					
	Sex		Age		Malocclusion	
	F	*P* value	F	*P* value	F	*P* value
PC1 - Symmetric component	0.331	0.566	0.027	0.871	1.570	0.212
PC2 - Symmetric component	8.370	0.005*	0.232	0.631	0.693	0.503
PC3 - Symmetric component	0.662	0.418	2.320	0.131	0.164	0.849
PC4 - Symmetric component	3.440	0.066	0.002	0.967	0.812	0.447
PC5 - Symmetric component	2.910	0.091	7.520	0.007*	3.010	0.053
PC6 - Symmetric component	0.025	0.876	6.980	0.009*	0.153	0.858
PC1 - Asymmetric component	0.837	0.362	0.602	0.439	0.618	0.541
PC2 - Asymmetric component	0.565	0.454	3.480	0.065	1.010	0.368
PC3 - Asymmetric component	0.061	0.805	0.617	0.434	1.430	0.244
PC4 - Asymmetric component	5.270	0.024*	0.795	0.374	1.280	0.282
PC5 - Asymmetric component	0.046	0.830	2.150	0.145	0.035	0.966
PC6 - Asymmetric component	0.135	0.714	0.167	0.684	0.808	0.449
Symmetric Log centroid size	14.300	<0.001*	0.946	0.333	0.062	0.940
Asymmetric Log centroid size	0.079	0.779	0.014	0.907	2.040	0.135
Mahalanobis shape FA scores	0.242	0.624	6.490	0.012*	2.970	0.056
Condylar volume (right and left)	18.600	<0.001*	1.450	0.231	2.180	0.118
Right-left condylar volume difference	0.327	0.568	0.379	0.539	0.175	0.839

PC - principal component, log - natural logarithm, FA - fluctuating asymmetry.
* Indicates statistical significance.
